# Cloud‐Based Control System with Sensing and Actuating Textile‐Based IoT Gloves for Telerehabilitation Applications

**DOI:** 10.1002/aisy.202400894

**Published:** 2025-02-17

**Authors:** Kadir Ozlem, Cagatay Gumus, Ayse Feyza Yilmaz, Asli Tuncay Atalay, Ozgur Atalay, Gökhan Ince

**Affiliations:** ^1^ Faculty of Computer and Informatics Engineering Computer Engineering Department Istanbul Technical University 34469 Istanbul Türkiye; ^2^ Faculty of Textile Technologies and Design Textile Engineering Department Istanbul Technical University 34437 Istanbul Türkiye

**Keywords:** cloud computing, machine learning, remote rehabilitation, soft robotics, textile sensors and actuators

## Abstract

Remote manipulation devices extend human capabilities over vast distances or in inaccessible environments, removing constraints between patients and treatment. The integration of therapeutic and assistive devices with the Internet of Things (IoT) has demonstrated high potential to develop and enhance intelligent rehabilitation systems in the e‐health domain. Within such devices, soft robotic products distinguish themselves through their lightweight and adaptable characteristics, facilitating secure collaboration between humans and robots. The objective of this research is to combine a textile‐based sensorized glove with an air‐driven soft robotic glove, operated wirelessly using the developed control system architecture. The sensing glove equipped with capacitive sensors on each finger captures the movements of the medical staff's hand. Meanwhile, the pneumatic rehabilitation glove designed to aid patients affected by impaired hand function due to stroke, brain injury, or spinal cord injury replicates the movements of the medical personnel. The proposed artificial intelligence‐based system detects finger gestures and actuates the pneumatic system, responding within an average response time of 48.4 ms. The evaluation of the system further in terms of accuracy and transmission quality metrics verifies the feasibility of the proposed system integrating textile gloves into IoT infrastructure, enabling remote motion sensing and actuation.

## Graphical abstract

Using cloud computing technology, a telerehabilitation application allows patients to perform exercises remotely. Sensor data from a textile‐based IoT glove is processed in the cloud and transmitted as exercise commands to an actuating T‐IoT glove worn by patients. This novel e‐health solution harnesses AI and IoT technologies to eliminate travel time and enhance medical staff–patient interaction.

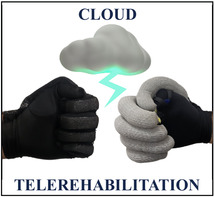

## 1 Introduction

The human hand plays a crucial role in our everyday activities, serving as a sophisticated and intricate tool that allows us to accomplish tasks accurately and effectively.^[^ ^1^ ^]^ Physical and neurological conditions such as stroke, burns, fractures, and ligament injuries can diminish finger range of motion and grip strength, while also increasing joint stiffness, significantly impacting individuals’ physical, psychological, and economic welfare, and substantially impeding their capacity to carry out activities of daily living.^[^ ^2^, ^3^ ^]^ Hand dysfunctions necessitate exercises assisted by medical staff to restore functionality, and success in musculoskeletal rehabilitation relies on numerous factors, including the timing, intensity, and frequency of the exercises.^[^ ^4^, ^5^, ^6^ ^]^

Many patients face obstacles that prevent them from accessing rehabilitation programs, such as financial constraints or difficulties in reaching therapy centers due to distance. Additionally, a lack of medical staffs restricts individuals from receiving the required rehabilitation for their physical enhancement.^[^ ^7^, ^8^ ^]^ Particularly during pandemics, medical staff‐dependent rehabilitation methods become especially vital.^[^ ^9^ ^]^ To overcome such hindrances, remote rehabilitation approaches have been developed within diverse Internet of Things (IoT) scenarios, involving the real‐time collection and processing of data through sensors.^[^ ^10^, ^11^ ^]^ This makes remotely guided training more accessible, affordable, and results‐driven^[^ ^12^ ^]^ thereby influencing the motivation of patients in therapy sessions.^[^ ^13^ ^]^

Hand exoskeleton robots, which mimic human hand movements and aid in rehabilitation by performing tasks such as grasp‐release exercises, are increasingly utilized in medical environments. Robots often controlled remotely through assistive robotic gloves, are also commonly employed in virtual reality applications.^[^ ^14^, ^15^ ^]^ Clinical research has demonstrated that stroke patients who participate in intensive repetitive movements through robotic hand therapy experience notable enhancements in hand motor functions. Furthermore, their central nervous system adeptly incorporates feedback from multiple senses to aid in motor learning when confronted with hand movements induced from various sources.^[^ ^16^, ^17^ ^]^

Studies concerning hand functionality can be categorized into two main areas: gesture recognition and motion control.^[^ ^18^ ^]^ Signals stemming from various sources have been investigated for the control of robotic hands, including activating buttons,^[^ ^19^ ^]^ physiological signals like ElectroEncephaloGram (EEG)^[^ ^20^ ^]^ and ElectroMyoGram (EMG),^[^ ^21^, ^22^ ^]^ voice commands,^[^ ^23^ ^]^ eye tracking,^[^ ^24^ ^]^ and even movements of the feet.^[^ ^25^ ^]^ For instance, vision‐based hand tracking utilizes cameras to monitor hand movements by employing machine‐learning (ML) techniques trained on extensive image datasets.^[^ ^26^, ^27^ ^]^ Another approach involves wearable hand tracking based on inertial measurement units (IMUs) and compasses. This method typically involves attaching accelerometers, gyroscopes, and magnetometers to the hand to measure its orientation, and then reconstructing the hand configuration by collecting angle data from each finger.^[^ ^28^ ^]^

The earlier developed sensor designs feature components that are difficult to utilize, intricate to manufacture, and susceptible to fragility.^[^ ^29^ ^]^ In vision‐based systems, a fundamental challenge arises when objects fall outside the camera's field of view (FOV), which remains unaddressed even with the application of machine‐learning techniques.^[^ ^30^ ^]^ For IMU and compass‐based systems, their vulnerability to magnetic field interference makes them impractical to use in close proximity to ferromagnetic objects.^[^ ^28^, ^31^ ^]^ Given these considerations, flexible, lightweight, and stretchable sensing technologies present a compelling solution as they provide excellent adaptability and can precisely capture the signals produced by fingers, facilitating safe interactions.^[^ ^32^, ^33^, ^34^ ^]^ Unlike rigid glove systems that may limit finger movement, soft gloves offer flexibility, allowing users with different finger sizes to maintain natural motion without any constraints.^[^ ^35^ ^]^

Assistive robotic glove systems have the capability to discern intricate hand features, gather low‐dimensional data, and achieve faster hand gesture recognition speeds.^[^ ^36^, ^37^ ^]^ Currently, rigid and soft robotic gloves have been designed to address the need for increased repetitions, utilizing various actuating systems such as pneumatic, hydraulic, electric, and tendon‐driven systems to provide mechanical power.^[^ ^38^ ^]^ Advanced robotic systems feature complex mechanisms that can produce the required forces and movements with precision for hand therapy. Yet, their intricate mechanical design, heaviness, and bulkiness present notable obstacles when developing hand exoskeletons.^[^ ^39^ ^]^ Soft robots’ physical flexibility presents an encouraging answer to the limitations faced by traditional robots in terms of safety and adaptability.^[^ ^40^, ^41^, ^42^ ^]^ Assistive devices, utilizing soft materials such as textiles^[^ ^34^, ^43^ ^]^ or elastomers,^[^ ^44^ ^]^ are inherently designed to be safe, lightweight, compliant, and non‐restrictive, facilitating prolonged wear.^[^ ^23^, ^45^, ^46^ ^]^

In this work, we present a novel IoT system for remote rehabilitation. This system utilizes a sensing textile‐based IoT glove (T‐IoT^[^ ^47^ ^]^ glove) as the master and a pneumatic actuating T‐IoT glove as the slave within a remote rehabilitation framework. In this study, we also validate the performance of the closed‐loop control system through the cloud computing system. Equipped with capacitive textile sensors on each finger, the T‐IoT glove is paired with a wireless transmitter incorporating an ML‐based recognition of the finger movements of the medical staff. The actuating T‐IoT glove mimics the finger motions captured by the sensors to assist individuals remotely with the required motions.

The sensing T‐IoT glove system integrates a Beetle bluetooth low energy (BLE) for the acquisition of sensor signals and transmitting them to the cloud via the medical staff's client program. The cloud system employed signal processing and ML analysis techniques to facilitate comprehensive telerehabilitation, requiring preprocessing and pattern identification to efficiently control the actuating T‐IoT glove. Cloud services are utilized to boost response time with minimal latency and fast transmission, as well as to reduce computational load.^[^ ^11^, ^13^ ^]^ This entails transferring the healthcare system's service module to the cloud, assigning computing resources according to users’ health conditions, and facilitating prompt interaction through transmission control protocol (TCP) and internet protocol (IP). The computation unit in the cloud performs gesture recognition with a 93.95% accuracy using ML techniques applied to the sensor data from the sensing T‐IoT glove. It generates real‐time control commands for the actuating T‐IoT glove. The actuating T‐IoT glove replicates the recognized actions, while computational tasks are efficiently handled by the cloud system, leaving only the communication tasks to run on edge devices with low computational resources.

To our knowledge, no existing system offers hand gesture recognition through a sensing T‐IoT glove and consequent remote control of the actuating T‐IoT glove utilizing machine learning over a cloud computing system all performed in real‐time. We anticipate that combining cloud computing with machine learning‐driven signal analysis will open up fresh opportunities, facilitating distinctive automatic detection, recognition, and prediction abilities in areas such as healthcare assessments and home‐based rehabilitation.

## 2 System Architecture and Material Designs

**Figure 1:**
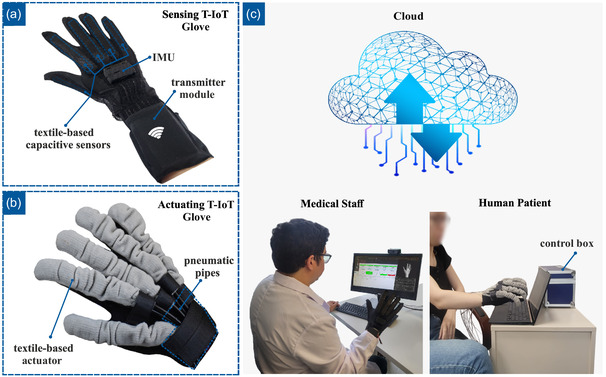
a) Sensing T‐IoT glove. b) Actuating T‐IoT glove. c) Telerehabilitation over the cloud with the medical staff wearing a sensing T‐IoT glove and the human patient wearing an actuating T‐IoT glove for telerehabilitation.

The structure of the proposed telerehabilitation system based on cloud computing is illustrated in **Figure** **1**. It comprises three main components: a sensing T‐IoT glove for gesture recognition, an actuating T‐IoT glove for rehabilitation, and an intermediate cloud computing architecture connecting the two gloves.

### 2.1 Design of the Sensing T‐IoT Glove

The sensing T‐IoT glove consists of five capacitive‐based textile sensors that are highly stretchable, making them fit the human hand. These highly sensitive capacitive sensors consist of two layers of knitted fabric coated with silver nanoparticles (Shieldex Medtex‐130, V Technical Textiles Inc., USA), which are utilized as upper and lower electrodes. Additionally, a silicon layer (Ecoflex 00‐30, SmoothOn Inc., USA) works as the dielectric material between them.

The change in capacitance in capacitive sensors is typically due to variations in the size of the sensor and can be utilized for detecting joint movements.^[^ ^47^ ^]^ Figure 1a illustrates the sensing T‐IoT glove, its sensors on the fingers, and the transmitter module on the wrist area. Silicon is cast on a conductive fabric with the desired thickness according to the required application, which also affects the performance of the sensor in terms of sensitivity and working range. The manufacturing methodology of these capacitive sensors, along with their working principle and characterizations are explained in detail in our previous work.^[^ ^48^ ^]^ Additionally, the characterization of the sensor with finger movement is provided in Section S1.1, Supporting Information.

After cutting the electrodes into the desired shapes using a laser cutting machine, particularly along the sides of the fingers, the silicon was cast. Following the silicon casting, these layers were merged and left to cure in an oven. Once shaped, the sensors were carefully positioned over the fingers of the glove. Connections with the Beetle BLE (DFRobot, Shanghai, China) development board and MPR121 capacitive sensor controller (Adafruit, New York, USA) were established using thermoplastic polyurethane (TPU)‐coated conductive yarns. This approach ensures that the flexibility of the sensing T‐IoT glove is not compromised and prevents the yarns from getting into contact with each other, thus minimizing the risk of short circuits and reducing parasitic capacitance.

### 2.2 Design of the Actuating T‐IoT Glove

The key components of the proposed actuating textile‐based IoT glove are illustrated in Figure 1b. The actuating T‐IoT glove consists of textile‐based knitted actuators capable of extending and bending upon applied force. The creation of such anisotropy is made possible by arranging the dorsal and plantar surfaces of the produced shells of the actuators to lead to localized expansion of fabric and actuator bending motion. This arrangement of knit loops and courses per centimeter in the computerized knitting machine (SHIMA SEIKI) controls needle yarn carriers simultaneously to create desired patterns. Another promising advantage of using computerized knitting during the production of actuators is eliminating the burdensome, time‐consuming steps of producing actuators via the cut‐and‐sew approach, which requires manual assembly.

The machine we employed allows for controlled mass production and reduces variation in products caused by manual assembly, meeting the demands of the health sector. Within these actuators, TPU sheets (Stretchlon 200, Fibre Glast) are utilized to create flexion and extension air pouches sealed by welding (impulse sealer PCS 300, Brother). The actuators are then mounted onto a base and worn using Velcro finger cuffs. Each bladder was manufactured by laser cutting two identical rectangles measuring 17 × 2.5 cm. The pneumatic system has been designed to provide forces to the actuators that will lead to their inflation, enabling the required action through fingers that are detected, processed, and conveyed via the sensing T‐IoT glove and cloud computing. Eventually, the actuating T‐IoT glove provides safer interactions with patients, thanks to the inherent lightweight and compliant nature of textile products. As these components communicate with each other via the Internet using IoT protocols, it ultimately causes the patient's hand to close and open during the therapy session. Detailed information about the material and methodology can be found in ref. [43]. Unlike previous studies, the effect of the air pressure applied to the actuator on the bending of the finger is shared in Section S1.2, Supporting Information.

## 3 Data Processing

**Figure 2:**
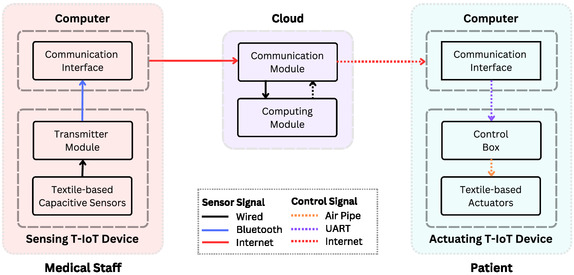
System architecture of sensor and control signals transmission.

The proposed system consists of three main components: the sensing T‐IoT glove, the actuating T‐IoT glove, and the cloud. **Figure** **2** illustrates the sensor and control signal transmission architecture of the proposed system. The exercise movements performed by the medical staff. The sensing T‐IoT glove captures the medical staff's finger movements via the capacitive sensors and transfers these data to a computer via Bluetooth, and then to the cloud in real time via the Internet. The cloud system processes the sensor data from the sensing T‐IoT glove using signal processing and machine learning techniques to generate appropriate control signals for the actuating T‐IoT glove. In addition to computing services, it also establishes a communication infrastructure for data transfer between the sensing and actuating T‐IoT gloves.

The actuating T‐IoT glove worn by the patient is responsible for executing the exercise movements received from the medical staff.

### 3.1 Data Processing in the Sensing T‐IoT Glove

The sensing T‐IoT glove consists of five textile‐based capacitive strain sensors and a transmitter module. The strain sensors are placed on the fingers. Depending on the movement of the fingers, there is a change in capacitance due to the mechanical alteration in the length and thickness of the sensor. Because of this mechanical effect, the capacitance increases when a finger is in a closed position and decreases when it is in an open position.

The transmitter module consists of a Beetle BLE, an MPR121 capacitive sensor controller, and a 3.7 V LiPo battery. The MPR121 capacitive sensor controller is used to measure the capacitance of the textile‐based capacitive strain sensors. One end of each sensor is connected together and attached to the ground of the module, while the other ends are connected to the electrode inputs. The module charges the sensors by providing the configured current value to each sensor individually for the assigned duration. The charge stored in the capacitance sensor is calculated as follows:

(1) Q=I⋅T

where *Q* is the stored charge, *I* is the assigned current value, and *T* is the charging time. The stored charge creates a voltage on the capacitive sensor, which is calculated as follows:

(2) Q=C⋅V

where *C* is the capacitance value of the sensor, and *V* is the voltage value resulting from the stored charge. At the end of the charging period, the capacitive sensor controller measures this voltage value via electrodes accessed through I2C. The values of *I* and *T* are determined during the configuration stage, and since the value of *V* can be obtained from the module, the capacitance value of the sensor is calculated as follows:

(3) I⋅T=C⋅V

(4) $$C=\frac{I\cdot T}{V}$$

The minimum capacitance and supply voltage of the sensors are used to configure the values of *I* and *T*. Using Equation (4), I⋅T can be determined as follows:

(5) $$I\cdot T<C_{\text{min}}\cdot V_{\text{dd}}$$

where $C_{\text{min}}$ is the minimum capacitance value, and $V_{\text{dd}}$ is the supply voltage of the capacitive sensor controller. Additionally, the capacitive sensor controller incorporates a two‐level filter structure to denoise the capacitance measurements.

The Beetle BLE, which is an Arduino‐based development board, collects the voltage values generated by the strain on the sensors in all five fingers at a sampling frequency of 50 Hz using the I2C protocol. These collected data are transmitted in real‐time to a computer via Bluetooth. In the computer environment, a developed interface receives the data and transfers it to the cloud via a TCP socket connection established with the cloud.

### 3.2 Data Processing in the Cloud Architecture

The cloud architecture has two primary tasks: communication and computation. In the communication task, data from finger sensors sent by medical staff is received via a socket connection. These data are added to the buffer associated with the user. Subsequently, through feature extraction, normalization, and ML, the raw sensor data for each finger is transformed into four control states: “Opening”, “Open”, “Closing”, and “Close”. These control signals are then transmitted in real‐time to the actuating T‐IoT glove on the patient site via a socket connection.

The communication module in the cloud serves as a socket server that connects the patient and the medical staff. Users and medical staff connect to the system using tokens created specifically for them. These tokens contain the role and meeting information of the users, which is used to match them accordingly. Information from the medical staff's sensing T‐IoT glove is processed and sent as control signals to the patient's actuating T‐IoT glove.

Additionally, the communication module transfers sensor data from the medical staff to the computation module. The computation module can be configured within the same cloud device or across different cloud devices. To minimize network latency, it is recommended to have the computation module within the same device.

The computation module is responsible for processing the sensor data transmitted by the communication module. As soon as a medical staff connects to the system, the computation module creates an object for the therapy service. This object contains a buffer for storing real‐time data for each finger, calibration parameters specific to the fingers, and ML models. The buffer is used to extract time series features from the real‐time incoming data.

To use ML, raw data, as well as the first, second, and third derivatives of the data for each finger, are extracted. These features are then normalized using a standard scaler.^[^ ^49^ ^]^ To determine the parameters of the standard scaler, the medical staff is expected to perform several consequent grasp movements for calibration when connected to the system. After this period, the standard deviation (SD) and mean values of features for each finger are recorded for the standard scaler, and the same calibration parameters are used throughout the operation.

The extracted features are converted into control signals using pre‐trained classification models for each finger. In this study, models were trained using ML methods such as logistic regression (LR), decision tree (DT), K‐nearest neighbors (KNN), multilayer perceptron (MLP), and XG‐Boost (XGB). The grid search method was used for hyperparameter tuning to improve the performance of the models. The trained models are stored in the cloud and are loaded into their respective objects each time a new medical staff connects to the system.

The real‐time predictions of finger states made by the ML models are transmitted to the communication module, to be further sent to the patient in real time via a socket connection. The use of socket connections in the communication module establishes a seamless end‐to‐end data bridge between sensing and actuating T‐IoT devices. This prevents the need to repeatedly establish connections for each data transmission, as required in systems utilizing SOAP or REST, avoiding repeated handshaking operations.

Furthermore, the actuating T‐IoT glove application does not need to continuously query the server to check if a control signal has been generated. When a signal is generated, it is directly transmitted to the device by the server.

### 3.3 Control of the Actuating T‐IoT Glove

The actuating T‐IoT glove operates on the principle of fluidic drive. Pressurized air created by an air pump reaches the bladders through valves. There are two bladders for each finger. When air is supplied to the upper bladder, the actuator bends; when air is supplied to the lower bladder, the actuator extends. When pressurized air is given to one bladder in a finger, the air release valve of the other bladder opens, and the air inside is expelled due to the pressure created by the inflation of the other bladder. The working principle and control scheme of the valves are addressed in detail in ref. [43].

The client application on the patient's computer facilitates the connection between the cloud and the actuating T‐IoT glove. The control signal generated by the cloud is sent to the patient's computer. The client application transmits the control signals to the microcontroller of the actuating T‐IoT glove via the serial port. The microcontroller initiates the task of opening or closing the respective finger whenever there is a change in the current finger state.

To detect the opening and closing actions of the fingers, the microcontroller is equipped with a pressure sensor for each bladder. Before starting rehabilitation, the open and closed positions for each finger are set via the client screen, and the threshold values for the bladders are determined. When a bladder reaches its threshold value in the opening or closing state, the movement is considered complete. The microcontroller controls each finger individually. If multiple fingers are moving simultaneously, the pressure in each finger is controlled separately. If one finger completes its movement, pressurization for that finger stops while the others continue their movements.

## 4 Results

In the first section, the performance analysis of the sensing T‐IoT glove was conducted by examining the accuracy of models trained using different ML methods on the created dataset. Subsequently, the trained models were integrated into the cloud, and their end‐to‐end operational performance was evaluated. In these tests, parameters such as time performance, resource usage, and network bandwidth usage were examined to analyze the impact of different ML models on cloud computing.

### 4.1 Results on Accuracy

For the control of the actuating T‐IoT glove, data was collected from 12 test subjects using the sensing T‐IoT glove, with ≈161 min of data for each finger. During data collection, the test subjects labeled the data as “Opening”, “Open”, “Closing”, and “Close”. The dataset collected from test subjects is divided into training (75%) and test (25%) sets. Using different ML methods models were trained for each finger, and the average test results are presented in **Table** **1**.

**Table 1:** Prediction performance of different classifiers.

Classifier	F1 scores of individual finger models					Average performance of all models			
	Thumb	Index	Middle	Ring	Pinkie	Accuracy	Recall	Precision	F1score
LR	79.05	87.97	89.45	88.66	88.01	87.32	85.89	87.65	86.63
DT	90.90	94.13	94.06	94.09	94.08	93.93	93.49	93.42	93.45
KNN	88.38	92.43	92.57	92.56	92.48	92.24	91.86	91.52	91.68
MLP	83.63	90.67	91.50	91.02	90.69	90.01	89.62	89.43	89.50
RF	**91.53**	**94.56**	**94.51**	**94.52**	**94.64**	**94.40**	**93.93**	**93.98**	**93.95**
XGB	91.36	94.39	94.46	94.43	94.60	94.30	93.85	93.85	93.85

When the scores of models trained with different machine learning methods were examined, the random forest (RF) method emerged as the most successful across all fingers. The RF models achieved a performance of 91.53 on the thumb and above 94.50 on the other fingers. The lower performance on the thumb is thought to be due to the thumb traveling a shorter distance than other fingers while closing, those moving more quickly, especially in actions involving multiple fingers moving simultaneously.

When compared with other methods based on F1 score, the XGBoost (XGB) and decision tree (DT) models achieved performances close to that of the RF models, with differences of 0.10% for XGB and 0.50% for DT. K‐nearest neighbors (KNN) showed a 1.77% lower performance, multi‐layer perceptron (MLP) showed a 4.45% lower performance, and logistic regression (LR) showed a 7.32% lower performance. It was observed that tree‐based models are more successful in solving the problem with the collected dataset.

**Figure 3:**
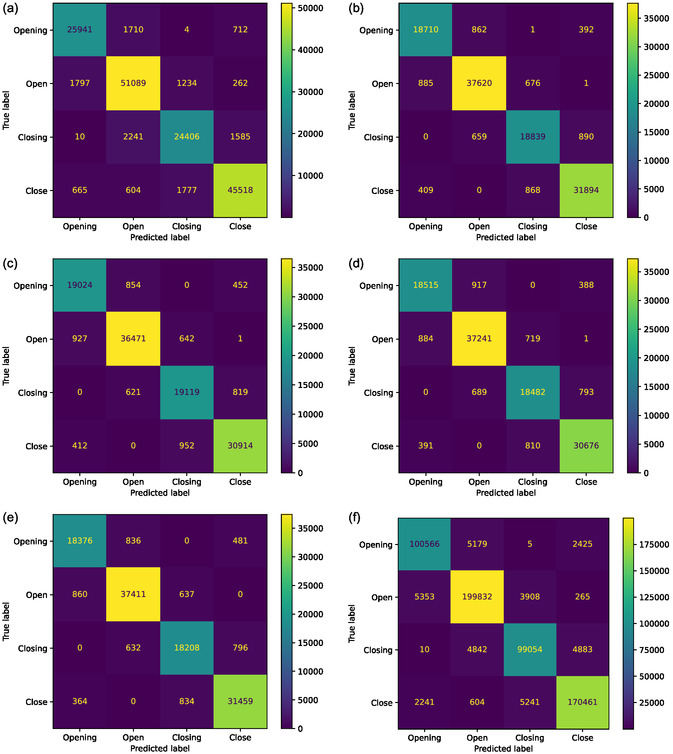
Confusion matrices of random forest classifier for different fingers. a) Thumb finger; b) index finger; c) middle finger; d) ring finger; e) pinkie finger; f) overall results.

**Figure** **3** shows the confusion matrices for the most successful model results, which belong to the RF models. Figure 3a–e present the confusion matrices for models trained separately for each finger. Figure 3f provides the combined results of models trained for each finger using the RF method. The confusion matrices for models trained using other classifiers are provided in Figure S6–S10, Supporting Information. When the confusion matrices are examined, it is evident that the majority of the data, as expected, lies on the diagonal of the matrices. The mispredictions are generally concentrated in states that transition into each other. In the dataset, the states progress cyclically as “Open”, “Closing”, “Close”, “Opening”, and “Open”. Given that transitions between states posed similar challenges during human labeling, it is understandable that the models also struggle with these predictions. Misclassifications are minimal for labels with distinct states in between, such as “Closing” to “Opening” or “Open” to “Close”.

### 4.2 Results on Time Performance

The developed cloud system provides real‐time computation and data communication between medical staff and their patients. Therefore, the timing performance of the system is of significant importance. To measure the system's timing performance, the following metrics were used: arbitration time, latency, queuing delay, execution time, jitter, and total response time.

**Figure 4:**
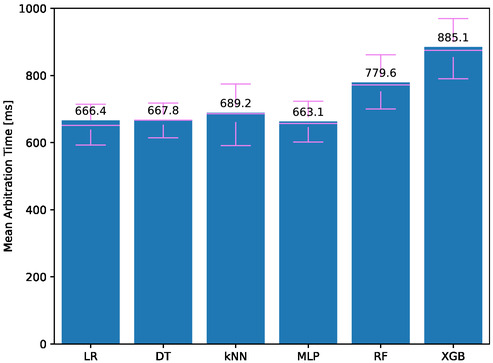
Arbitration time for different classifiers.

**Figure** **4** shows the preparation time of the system for different classifiers when a medical staff initiates a therapy request. The models for the classifiers are trained and saved to the system's computing module when it is first started. When a new medical staff starts a session, the saved model is loaded, and the necessary preliminary preparations for signal processing and feature extraction are made. Once these preparations are completed, the system notifies the medical staff and the patient that it is ready through the client program.

In the system, except for the type of trained model, all procedures such as signal processing and feature extraction are the same for all models. Therefore, classifiers with larger model sizes have longer arbitration times due to the length of file reading operations. The lowest average arbitration time was observed in tests using MLP at 663.1 ms, while the highest was observed using XGB at 885.1 ms. Models with multiple tree structures also had high arbitration times. Additionally, the system prepared with kNN showed high arbitration times because, instead of using a mathematical equation, the entire training set is loaded, and predictions are made based on the proximity to the training elements. When examining the results, even the longest time is under 1 s, so the arbitration times for all models are quite low. When a medical staff connects to the system, he/she can start receiving computation services from their dedicated system within 1 s.

**Figure 5:**
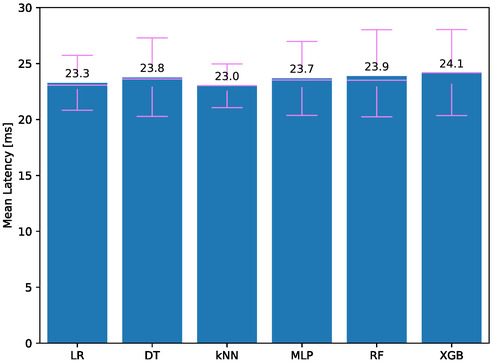
Latency for different classifiers.

**Figure** **5** shows the latency scores for therapies using different classifiers. The lowest average latencies are from KNN and LR, with scores of 23.0 and 23.3 ms, respectively. The other classifiers have latencies above 23.5 ms. Although the other models have slightly worse results, they can still send their data to the computation module with a delay of up to 25 ms.

**Figure 6:**
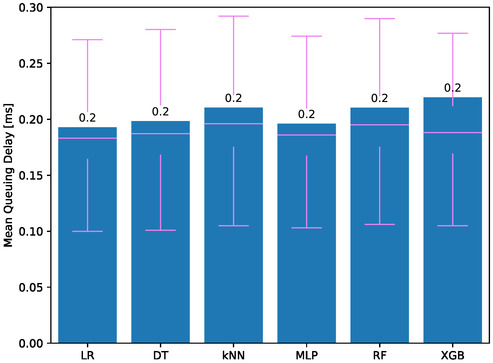
Queuing delay for different classifiers.

**Figure** **6** presents the average queuing delays for therapies using different classifiers. All models have very low and similar queuing delays of 0.2 ms, starting the data processing promptly. In addition to the low mean queuing delay, the standard deviation appears relatively large when compared to this value. Some computation requests experience a queuing delay of 1–2 ms, which is typically minimal. This slight delay occurs either because the previous task has not yet finished or because the processor is busy with other tasks. Since the majority of computation requests start processing without any waiting time, the average queuing delay is significantly low. The combination of a low mean value and relatively larger queuing delays contributes to the appearance of a high standard deviation. However, the low average queuing delay actually indicates that most of the system operates without waiting in the queue.

**Figure 7:**
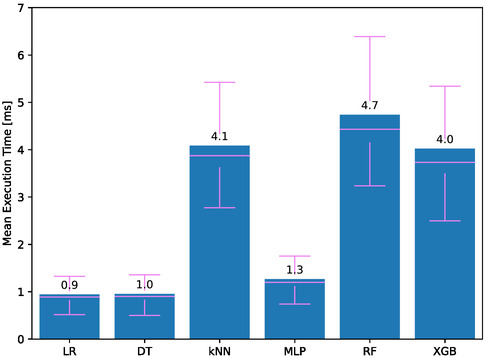
Execution time for different classifiers.

**Figure** **7** shows the time taken to process incoming sensor data and make state predictions for each finger. The lower the execution time, the more therapies the system can handle simultaneously. Therefore, execution time is one of the most important metrics for the system. According to the results, LR and DT therapies have the lowest execution time with scores of 0.9 and 1.0 ms, respectively.

**Figure 8:**
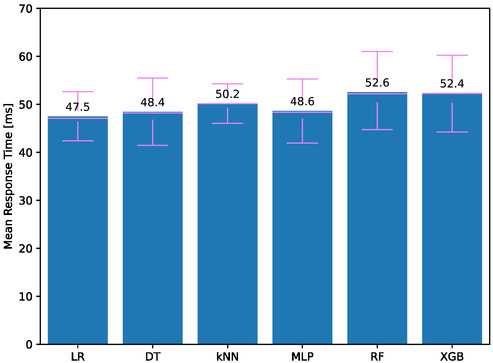
Total response time for different classifiers.

**Figure** **8** displays the average response times for therapies using different classifiers. The best scores are similar to previous results, with LR and DT models achieving 47.5 and 48.4 ms, respectively. The highest response times are observed with kNN, RF, and XGB. Overall, all models have an average delay of around 50 ms, which means they can provide real‐time services. However, for faster response times, it is recommended to use models trained with LR and DT methods.

**Figure 9:**
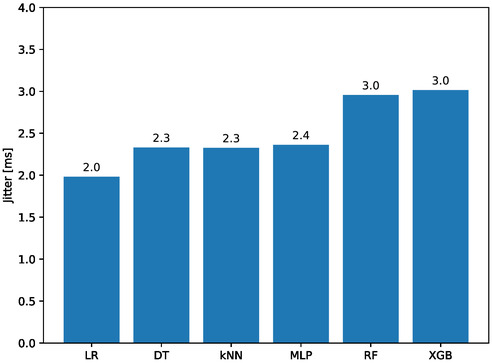
Jitter for different classifiers.

**Figure** **9** illustrates the jitter, or the variation in response time, for models trained with different classifiers. The lowest jitter is observed in models trained with LR at 2.0 ms, followed by DT and KNN at 2.3 ms. The highest jitter is seen in models trained with RF and XGB at 3.0 ms. High jitter can negatively affect synchronization between commands, potentially causing therapeutic movements to be shorter or longer than intended. However, since the obtained jitter values are relatively short compared to hand movements, they can be considered negligible.

### 4.3 Results on Resource Usage

In computational systems, resource utilization directly affects various time metrics such as response time, queuing delay, and execution time. The higher the resource usage, the longer the system will take to respond to new processes. Additionally, lower CPU consumption indicates that the system can serve more users and also results in lower cloud costs.

**Figure 10:**
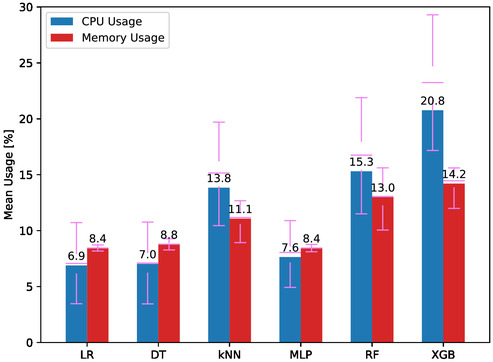
CPU and memory usage for different classifiers.

**Figure** **10** shows the resource consumption during therapy for models developed with different classifiers. The lowest CPU usage is observed with LR and DT models at 6.9% and 7.0%, respectively. For memory usage, the lowest values for LR and MLP are both 8.4%, while DT has a slightly higher minimum at 8.8%. The highest CPU usage is 20.8% and the highest memory usage is 14.2%, both for XGB. Given the lower CPU and RAM usage, models trained with LR, DT, and MLP are recommended as they can serve more users and are more cost‐effective.

### 4.4 Results on Network Bandwidth Usage

**Figure 11:**
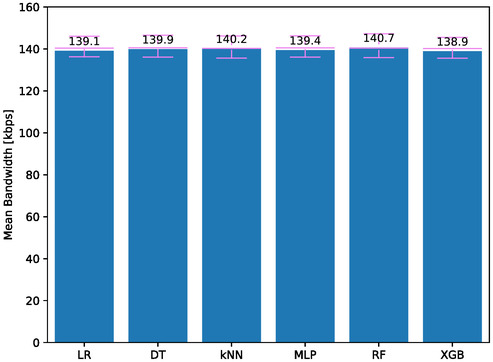
Network bandwidth for different classifiers.

Network bandwidth usage affects network traffic and can negatively impact network latency. However, in this study, since the incoming and outgoing data are the same for each model, similar network bandwidth usage is expected. **Figure** **11** shows the network bandwidth usage during therapy for models trained with different classifiers. The lowest value is 138.9 kbps for models trained with XGB, while the highest value is 140.7 kbps for models trained with RF. The difference between the lowest and highest values is observed to be 1.30%, with similar values across all models. Considering that cloud system network infrastructures operate at Gbps levels, the network bandwidth usage obtained is quite low.

## 5 Discussion

Despite extensive research on rehabilitation and assistive glove systems, many designs remain self‐driven and lack professional guidance. Our approach addresses this gap by integrating cloud‐enabled telerehabilitation with direct medical supervision, aligning therapy with patient‐specific goals, and embedding expert oversight in each session. As shown in Table S1, Supporting Information, our work uniquely combines remote rehabilitation capabilities with real‐time medical direction, offering a distinct advantage over prior systems that rely solely on patient‐driven functions.

Compared to existing literature works, the closest work to the proposed system is studied by Proietti et al.^[^ ^50^ ^]^ which combines soft robotics and cloud technologies. In their study, patients were trained with pre‐recorded tasks, and the system was controlled via a tablet. There was no direct interaction between the patient and the medical staff. Moreover, the cloud was used solely for monitoring the rehabilitation process. Another relevant study by Zhou et al.^[^ ^51^ ^]^ integrated sensors, actuators, and cloud technology, where a sensorized glove was used to control a rigid robotic hand. However, the primary focus of that study was robotic control rather than rehabilitation, and cloud technology was similarly employed only for monitoring. In contrast, our proposed system enables therapy sessions between patients and medical staff located in different locations while providing a safer solution for patients through the use of soft actuators. Additionally, our system leverages the cloud as a computational hub, reducing the computational load on edge devices.

In addition to the advantages offered by cloud technology, the developed actuating T‐IoT glove achieves a bending angle of 140° with pressurization of 50 kPa (Figure S3, Supporting Information). In other pneumatic soft robotic systems,^[^ ^12^, ^19^, ^45^, ^52^, ^53^, ^54^, ^55^ ^]^ bending of the actuators required pressures ranging from 70 to 345 kPa. Although the actuators developed by Zhu et al.^[^ ^56^ ^]^ was able to bend at 50 kPa exceptionally, they achieved a bending angle of only 90°. The proposed system demonstrates superior actuator performance, achieving a greater bending angle at lower pressure compared to other systems.

When examining the results of the developed system and trained models, RF is observed to be the most successful in terms of accuracy. However, it performs poorly in metrics such as arbitration time, latency, execution time, total response time, jitter, and CPU and Memory usage. Models trained with LR and DT methods have shown significantly better results in these metrics compared to RF, particularly in CPU usage, where they consume about half as many resources. It is estimated that a system using these methods could potentially serve approximately twice as many users as one using RF.

However, when examining the test results in terms of accuracy, there is a 7.32% difference in F1 score between models trained with LR and those trained with RF. This significant difference in prediction performance makes LR‐trained models less preferable compared to RF‐trained models. On the other hand, models trained with DT show only a 0.50% difference in F1 score and overall scores compared to RF models. Thus, by sacrificing just 0.50% in accuracy, the system can achieve lower response times and reduced resource consumption, which allows for serving more users and lowering resource costs as a trade‐off.

Upon examining the accuracy scores of the proposed system, an F1 score of 93.95 was observed. The reasons for mispredictions in the system include the inability of machine learning methods to fully distinguish transitions between states, variations in the anthropometric hand data of test subjects, and hand tremors during movements. The lower accuracy score for the thumb, compared to other fingers, is attributed to its shorter movement distance, leading to less variation in sensor data.

In the system's use for therapy, it is anticipated that medical staff will observe the patient's hand movements in real‐time through a third‐party video conferencing system. Since the proposed system is not intended for use in critical scenarios such as surgery, it can be argued that the system is more tolerant of trade‐offs between latency and accuracy.

## 6 Conclusion

This study has demonstrated the efficacy of a telerehabilitation strategy by utilizing cloud computing‐based IoT devices such as textile‐based sensing and actuating gloves for patients with hand impairments. The system developed is introduced into the field of remote healthcare to complement conventional therapy, aiming to overcome location hindrances, thereby enabling continuous improvement regardless of location. The application of advanced machine learning algorithms for interpreting real‐time hand movements, captured by the sensing T‐IoT glove and processed via cloud technology, enables the control of the actuating T‐IoT glove. The proposed system performed an average response time of 48.4 ms and an average accuracy of 93.45%.

Since our aim was to eliminate injury risks that might occur during conventional robotic therapy applications, due to the lightweight, soft attributes of textile materials, the system provides compliant and safe interactions. We believe that our approach will enhance the outcomes of hand therapy aimed at restoring neuromuscular function, thereby increasing the quality of life for people. Moreover, the system architecture developed for cloud computing can be implemented not only in IoT systems for rehabilitation but also in other applications such as remote medical surgeries or virtual/augmented reality applications. In future work, the actuating T‐IoT glove can be transformed into a holistic robotic T‐IoT glove by incorporating both sensing and actuating capabilities in a single body with a reciprocal function. Furthermore, clinical and human‐computer interaction tests with real patients are also planned for the system.

## 7 Experimental Section

In this section, the test conditions for evaluating the proposed telerehabilitation system are described. Details about data collection and labeling, demographic information of the test subjects, as well as the experimental setup, experimental scenarios, and evaluation criteria are provided in detail.

7.1

7.1.1

##### Dataset and Labeling

A machine learning‐based control technique is applied for controlling the actuating T‐IoT glove using the data from the sensing T‐IoT glove. Machine learning is used to detect the states of the fingers, and the actuating T‐IoT glove moves according to these states. To utilize machine learning techniques, appropriate data collected under suitable conditions is necessary. In this study, data is collected from 12 test subjects with an average age of 27.3 years, average anthropometrics hand data^[^ ^57^ ^]^ of hand length of 17.1 ± 1.3 cm, and hand breadth of 7.9 ± 0.5 cm. Demographic information of the test subjects is provided in **Table** **2**. Eight of the test subjects are female and four are male. Excluding the data collected for calibration in the dataset, an average of 161 min of data per finger has been used for machine learning. This study was approved by the Ethics Committee of Istanbul Technical University Human Medical and Engineering Research (SM‐INAREK‐2021‐03). Written informed consent was obtained from all participants prior to data collection.

**Table 2:** Test subject demographic information.

Test subject	Age	Sex	Height [cm]	Weight [kg]	Anthropometrics hand data	
					Hand length [cm]	Hand breadth [cm]
1	32	Male	172	92	19.2	8.7
2	25	Female	157	53	15.4	7.5
3	28	Female	167	50	17.5	7.5
4	31	Male	173	70	18.0	8.5
5	26	Female	164	55	18.5	8.0
6	31	Female	161	60	15.0	7.5
7	22	Male	181	67	17.0	8.5
8	22	Male	183	62	17.0	8.0
9	23	Female	167	69	18.0	8.5
10	40	Female	168	65	17.0	7.0
11	21	Female	163	69	16.0	8.0
12	27	Female	158	51	16.0	7.5
Average ± SD	27.3 ± 5.5	F/M: 8/4	167.8 ± 8.2	63.6 ± 11.5	17.1 ± 1.3	7.9 ± 0.5

**Figure 12:**
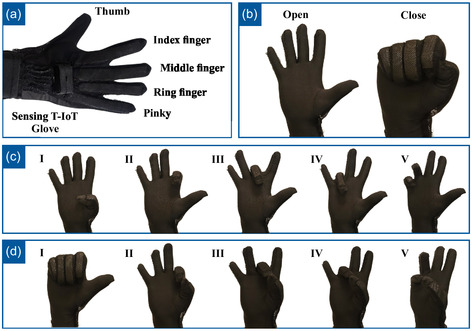
a) Sensing T‐IoT glove and its fingers. b) Sensing T‐IoT glove calibration and fist movement: open hand and close hand. c) Single finger closing and opening: I) Thumb, II) Index, III) Middle, IV) Ring, and V) Pinkie. d) Motions with a combination of fingers closing and opening: I) Open thumb and other fingers close, II) Cylindrical pinch with thumb and index, III) Cylindrical pinch with thumb and middle, IV) Cylindrical pinch with thumb and ring, V) Cylindrical pinch with thumb and pinkie.

A procedure for data collection was laid down with medical experts the sensing T‐IoT glove shown in **Figure** **12**a was worn to the right hand. At the beginning of the test, subjects were instructed to open and close their hand as shown in Figure 12b several times. This data was used for the calibration of normalization parameters specific to each test subject. All data, including calibration data, was saved into separate files during experiments.

After the calibration stage, subjects were asked to perform different movements. The experimenter shows the movement to the test subject at the beginning of each new movement. After the experimenter confirms that the test subject has correctly performed the movement, the recording of the test is initiated. Each movement was repeated sequentially 30 times. Subjects were instructed to wait around one second in the “Close” and “Open” states of the movements. They were allowed to perform movements at any speed they favored. During the movements, subjects were also asked to label active fingers using buttons. A five‐second break was given to the subject before each new movement.

**Figure 13:**
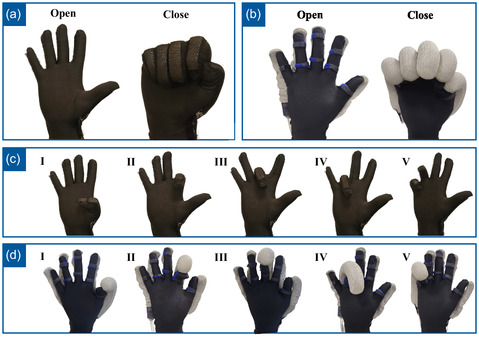
a) Sensing T‐IoT glove calibration and fist movement: open hand and close hand. b) Actuating T‐IoT glove calibration and fist movement: open hand and close hand. c) Sensing T‐IoT Glove single finger closing and opening: I) Thumb, II) Index, III) Middle, IV) Ring, and V) Pinkie. d) Actuating T‐IoT glove single finger closing and opening: I) Thumb, II) Index, III) Middle, IV) Ring, and V) Pinkie.

First, the test subjects performed a fist gesture, fully opening and closing the hand as depicted in Figure 12b. Afterwards, they individually opened and closed each finger, as shown in Figure 12c. Following them, they opened the thumb while closing and opening the other four fingers 30 times (Figure 12d,I). Finally, they sequentially touched the rest of the fingers with the thumb in cylindrical pinch movements, completing the data collection process as shown in Figure 12d,II–V. The gestures performed by the sensing T‐IoT glove and their corresponding gestures executed by the actuating T‐IoT glove are depicted in **Figure** **13**. Figure 13a,b presents the “Open” and “Close” states of the T‐IoT gloves, which are critical for grasping and releasing motions. Figure 13c,d illustrates the necessary individual finger movements essential for single‐finger exercises. A demonstration of these gestures can be observed in Supporting Video S1, Supporting Information.

A GUI is designed for the experimenter (Figure S4, Supporting Information). This interface allows for the observation of real‐time analog data from the sensors, along with cycle count, test duration, active fingers, and the current state of the active fingers. The test name to be conducted by the test subject is selected through this GUI, and the active fingers are determined based on the test to be performed. The test can be initiated and stopped within this GUI. Additionally, in the developed interface, sensor data from the 5 fingers and the user's labels are displayed in real‐time (Figure S5, Supporting Information). The experimenter monitors the data collection process, identifies any issues, and notes errors. After the experiments, the collected data are labeled manually as “Opening”, “Open”, “Closing”, and “Close”.

##### Experimental Setup

In the proposed system, real‐time data from the sensing T‐IoT glove worn by the medical staff is converted into control signals for the actuating T‐IoT glove worn by the patient. This setup provides telerehabilitation infrastructure for medical staff‐patient interactions at separate locations.

Performance testing of the developed system utilized a traffic generator and cloud devices as follows. ➀ Traffic generator and receiver: Dell Core i7‐8550U CPU @1.80 GHz, 32 GB DDR4 RAM, 240 GB SSD, and Windows 11 Enterprise 64‐bit). Software: Node.js v18.15.0 and Python 3.9.13; ➁ Cloud device: Digital Ocean VPS (2x Dedicated Premium Intel CPU, 8 GB RAM, 50 GB SSD, Cloud Location: Frankfurt/Germany).

Traffic generator and receiver ➀ replicates the clients of the sensing and actuating T‐IoT gloves. To prevent synchronization issues that may arise from using different devices, tests were conducted solely on one traffic generator.

Cloud device ➁ provides computational services and communication infrastructure to IoT devices. It processes sensor data coming from the medical staff's application, converts it into control signals, and then sends the processed data to the patient's application. In this way, it acts as a bridge between the patient and the medical staff.

During the experiments, both applications are initiated simultaneously and connected to the cloud. At this stage, the medical staff's application informs the cloud about the machine learning model it intends to use during the connection. The cloud application loads the registered model, creates necessary buffers for sensor data, and generates objects associated with them.

After the preparations are completed the medical staff's application receives a notification. Subsequently, the medical staff's application starts to send the previously recorded data sampled at a frequency of 50 Hz. This procedure is repeated similarly for 11 different movements for each test subject. Tests for all machine learning models are repeated using the same procedure. The reason for using recorded data in the performance tests of the proposed system is to ensure that the tests for all models are conducted under the same conditions.

For the evaluation of machine learning performance, the data was randomly split into 75% training and 25% testing sets. The same training and testing sets were used across all machine learning models. Hyperparameter tuning was performed using the grid search method. The parameters of the classifiers are given **Table** **3**, and the rest of the parameters are set as default.

**Table 3:** Model parameters of the classifiers.

Classifier	Parameter	Value
LR	Inverse of regularization strength	100
DT	The minimum number of samples required to split an internal node	8
KNN	Number of neighbors	20
MLP	Activation function for the hidden layer	tanh
RF	The number of trees in the forest	20
XGB	Number of gradient boosted trees	25
	Maximum tree depth for base learners	18

##### Evaluation Criteria

Accuracy analysis has been conducted for the performance of the T‐IoT devices and trained models. Criteria such as time characteristics, cloud assessing resource usage, and network bandwidth usage have been used to examine the performance of the cloud computing and communication modules.

Accuracy, recall, precision, and F1 score metrics were used to evaluate the performance of the trained models in this study. The following equation was used for calculating accuracy:

(6) $$\text{accuracy}(y,\hat{y})=\frac{1}{N}\sum_{i=0}^{N}1({\hat{y}}_{i}=y_{i})$$

where *y*_*i*_ is actual label of the *i*‐th sample, ${\hat{y}}_{i}$ is predicted label of the *i*‐th sample, *N* is sample numbers.

The recall, precision, and F1 score metrics were calculated as follows:

(7) $$P_{c}=\frac{{\text{TP}}_{c}}{{\text{TP}}_{c}+{\text{FP}}_{c}}$$

(8) $$R_{c}=\frac{{\text{TP}}_{c}}{{\text{FN}}_{c}+{\text{TP}}_{c}}$$

(9) $$F1_{c}=\frac{2\cdot P_{c}\cdot R_{c}}{P_{c}+R_{c}}$$

where *P*_c_ is precision score for class *c*, *R*_c_ is recall score for class *c*, and $F1_{c}$ is F1 score for class *c*. ${\text{TP}}_{c}$ is true positive prediction of class *c*, ${\text{FP}}_{c}$ is false positive prediction of class *c*, and ${\text{FN}}_{c}$ is false negative prediction of class *c*. The final scores for precision, recall, and F1‐score, as well as the average performance of all models, were calculated using the macro‐average method as follows:

(10) $$\text{MacroScore}=\frac{\sum_{c=0}^{C}{\text{Score}}_{c}}{C}$$

where ${\text{Score}}_{c}$ is the score of the c‐th class and *C* is the number of the classes.

Time metrics are crucial for measuring real‐time performance. In this study, six time characteristics were defined as follows: 1) Arbitration time: It refers to the duration between the traffic generator initiating the connection to the cloud and the cloud completing the necessary preparations to be ready to provide services for the user. Once the cloud is ready to serve the user, it sends a notification, after which the traffic generator starts sending data; 2) Latency: It refers to the duration between the traffic generator sending the data and the data entering the computation queue; 3) Queuing delay: It refers to the time that a computation request waits in the queue before it can be processed; 4) Execution time: It refers to the entire process during which sensor data from five fingers undergo preprocessing and machine learning processes to convert them into control signals; 5) Total response time: It refers to the duration between the computation request being sent from the traffic generator, processed in the cloud, and reaching the robotic glove client as a control signal; 6) Jitter: It is the fluctuation in the total response time. It is calculated by determining the standard deviation of this duration.

**Figure 14:**
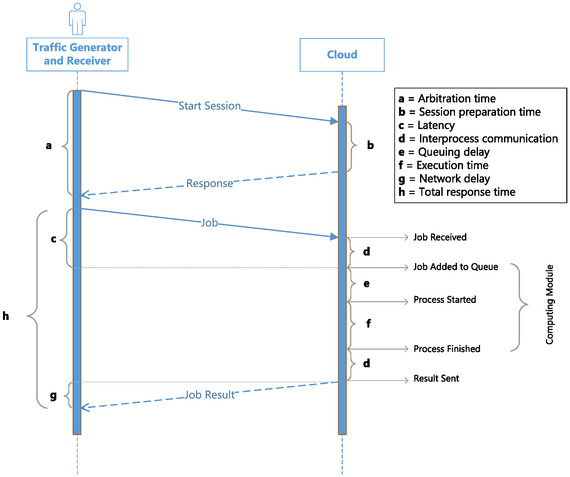
Sequence time diagram of arbitration and job request operation.

**Figure** **14** illustrates the time metrics on a sequence diagram. To calculate these time metrics, the traffic generator records a timestamp for each data transmission and reception. The difference between these two timestamp values represents the arbitration time (a) for the session start operation and the total response time (h) for job requests.

On the cloud side, timestamps are recorded for the following events in job requests: “Job Received,” “Job Added to Queue,” “Process Started,” “Process Finished,” and “Results Sent.” Using these timestamps, the interprocess communication time (d), queuing delay (e), and execution delay (f) metrics are calculated.

The latency (c) and network delay (g) metrics cannot be directly calculated because they involve different devices with potential temporal synchronization errors. For network delay, the round‐trip times in the network are assumed to be symmetric. By subtracting the time spent in the cloud (“Request Sent”‐“Job Received”) from the total response time measured by the traffic generator, the time the data spends in the network is determined. Half of this value is considered the network delay (g). Latency is calculated as the sum of network delay (g) and interprocess communication time (d) metrics.

Resource usage provides the RAM and CPU usage of the cloud device during computation and communication processes. Resource usage data is transmitted to the traffic generator at 1‐second intervals and stored. High resource usage in the system can lead to an increase in time characteristics, thereby reducing the real‐time capability of the system. Network bandwidth usage equals the total of incoming and outgoing data in real‐time on the cloud system. An increase in network bandwidth usage increases resource usage and consequently leads to delays in the system.

## Conflict of Interest

The authors declare no conflict of interest.

## Author Contributions

**Kadir Ozlem**: conceptualization (lead); data curation (lead); formal analysis (lead); investigation (lead); methodology (lead); resources (lead); software (lead); validation (equal); writing—original draft (lead); writing—review & editing (equal). **Cagatay Gumus**: conceptualization (equal); investigation (equal); methodology (equal); validation (equal); writing—original draft (equal). **Ayse Feyza Yilmaz**: conceptualization (equal); investigation (equal); methodology (equal); resources (equal); visualization (equal). **Asli Tuncay Atalay**: conceptualization (equal); methodology (equal); project administration (equal); resources (equal); supervision (equal); validation (equal); writing—review & editing (equal). **Ozgur Atalay**: funding acquisition (equal); methodology (equal); project administration (equal); supervision (equal); validation (equal); writing—review & editing (equal). **Gökhan Ince**: conceptualization (equal); funding acquisition (lead); methodology (equal); project administration (lead); supervision (lead); validation (equal); writing—review & editing (lead).

## Supporting information

Supplementary Material

## Data Availability

The data that support the findings of this study are openly available in GitHub‐TelerehabilitationGloves at https://github.com/kadirozlem/TelerehabilitationGloves, reference number 854780829.
