# Supplementary material for: Cloud‐Based Control System with Sensing and Actuating Textile‐Based IoT Gloves for Telerehabilitation Applications
Source: Adv Intell Syst. 2025 Feb 17;7(8):2400894. doi: 10.1002/aisy.202400894 (PMC12370170; doi:10.1002/aisy.202400894)
Supplement: Supplementary file 1 — Supplementary Material [file AISY-7-0-s001.zip › aisy1580-sup-0001-SuppData-S1.pdf]

Supporting Information

Cloud-based Control System with Sensing and Actuating Textile-based IoT Gloves for Telerehabilitation Applications

*Kadir Ozlem, Cagatay Gumus, Ayse Feyza Yilmaz, Asli Tuncay Atalay, Ozgur Atalay, Gökhan Ince**

1 Characterization of T-IoT Gloves

In this study, a Sensing T-IoT Glove was used to detect the finger movements of medical staff, while an Actuating T-IoT Glove was employed to move the patients' fingers. Unlike previous studies, the characterization of sensors and actuators is associated with finger motion, since in this study the focus is on finger movement. During the testing of both gloves, finger movements were detected using a label-based image processing technique.

In the tests, the index finger was used. Labels were placed on the Distal Phalanx (DP), Proximal InterPhalangeal joint (PIP), and MetaCarpoPhalangeal joint (MCP) of the index finger, as well as on the thumb side as shown in Figure S1(a). By using image processing techniques, the positions of these points were determined. The angle formed by the DP and MCP points at the PIP point was identified and symbolized as α.

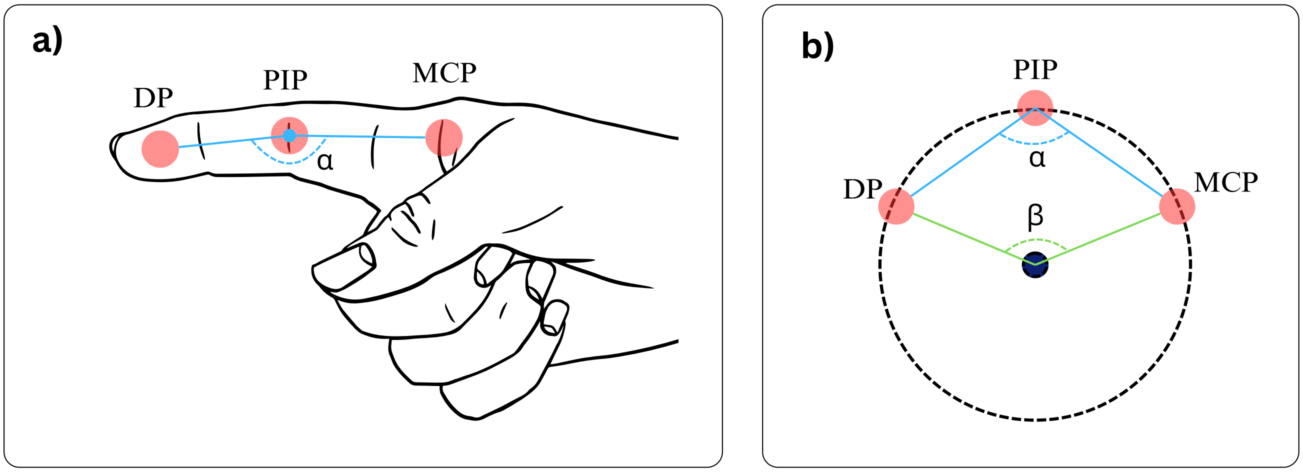

Figure S1: Label Configuration for Bending Angle Calculation of Finger Movement: (a) Label Placement on the Index Finger, (b) Bending Arc Representation of Label Positions.

The bending angle of the index finger (β) was defined as the angle of the arc formed by the bending of the finger (Figure S1(b)) and calculated as follows.

$$\beta = 360 - 2 \times \alpha \tag{1}$$

The bending angle ranges between 0° and 360°. A bending angle of 0° corresponds to a fully straight finger, also referred to as the "Open" position. An angle of 360° indicates full flexion or the "Close" position. However, physically achieving a complete 360° closure is impossible, as it would require the two labels to overlap.

In the finger bending angle experiments of both sensing and actuating T-IoT gloves, an 8-cycle test was conducted following 4 cycles of preliminary tests, which all involve consequent flexion and extension movements.

1.1 Characterization of Sensing T-IoT Gloves

In this test, the test subject was asked to open and close their finger at different speeds. The average closing time of the finger was 1.54 seconds, while the opening took 1.88 seconds in average. Including the average waiting times between each cycle, one complete cycle lasted approximately 6.06 seconds. The bending angle-capacitance graph for the index finger of the Sensing T-IoT Glove is shown in Figure S2. While the angle values range from 0.1° to 240.4°, the capacitance values vary between 147.6 pF and 160.1 pF. The closing movement produced a more linear result, whereas the opening movement resulted in a more curved pattern and had lower capacitance values compared to the closing movement. This discrepancy is thought to be due to the recovery behavior of the textile structure.

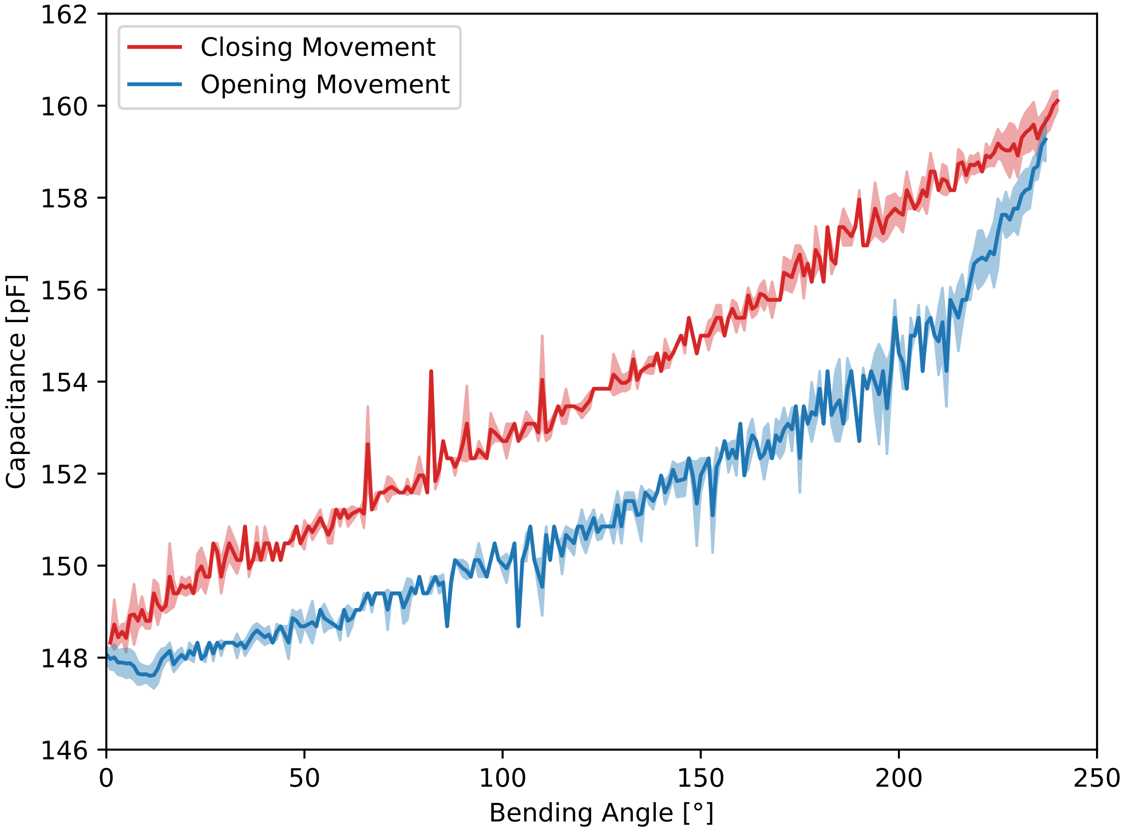

Figure S2: Capacitance Change of Textile-based Sensor During Index Finger Movement.

1.2 Characterization of Actuating T-IoT Gloves

In this test, the test subject holds his/her finger without applying any force, and the flexion bladder is pressurized up to 150 kPa for the flexion movement, while the extension bladder is used during the extension movement. When one bladder is pressurized, the exhaust valve of the other bladder is opened, allowing air to escape into the atmosphere.

The flexion movement took an average of 5.92 seconds, while the extension movement lasted 3.45 seconds. A waiting time of 1 second was applied between movements, resulting in an average cycle duration of 11.37 seconds. The actuator was operated slower than the sensing glove to minimize the risk of potential harm to the patient whereas the system is capable of operating at a faster rate.

Figure S3 shows the angle change of the index finger in response to pressure applied by the Actuating T-IoT Glove. The shared pressure data pertains to the flexion bladder, which is directly related to the flexion movement. The results show that the flexion movement exhibited a more linear response to the applied pressure, while the extension movement responded more slowly to pressure reduction. The pressure values ranged from 102.8 kPa (approximately atmospheric pressure) to 150.0 kPa. Correspondingly,

the angle values varied between 32.8° and 185.0°. It was observed that the finger's bending and extension movements had a slightly smaller range compared to a healthy person. This limitation is expected, as the Actuating T-IoT Glove contains exoskeletal actuators, which naturally restrict movement to some extent.

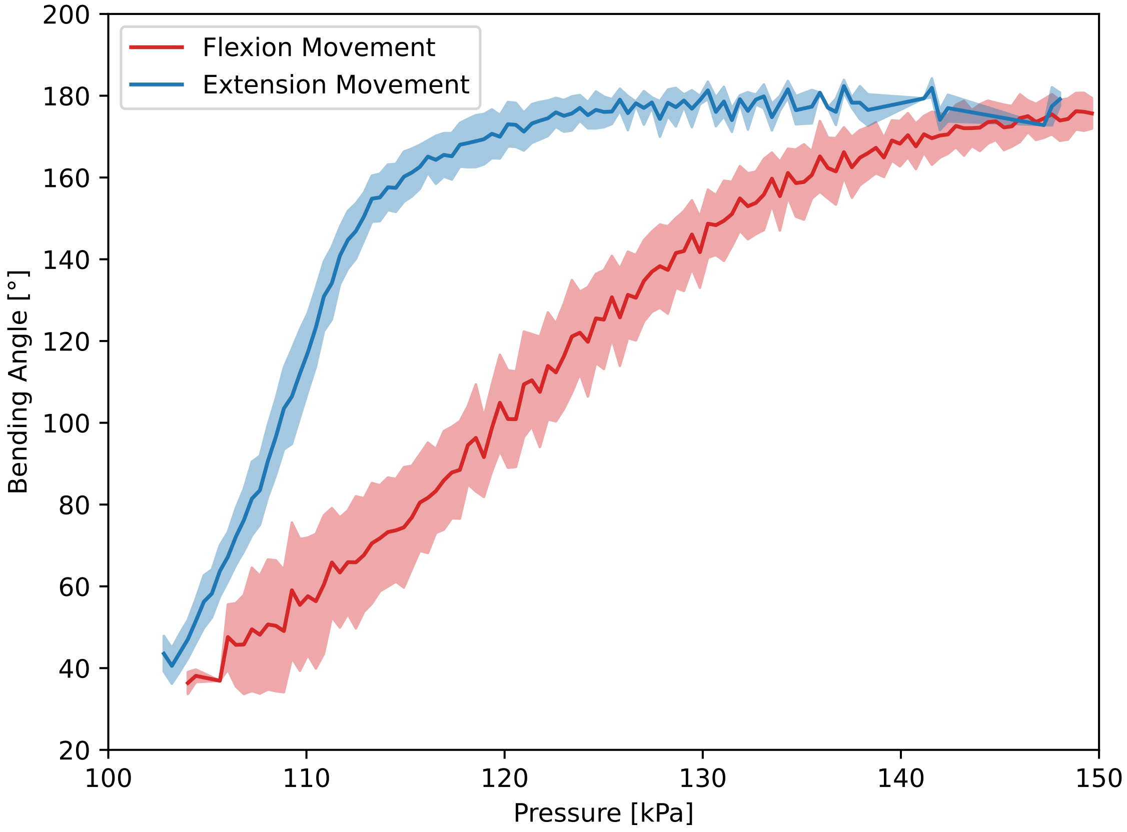

Figure S3: Change of Index Finger Bending Angle During Flexion and Extension Movement of Textile-based Actuator.

2 The Other Supporting Figures

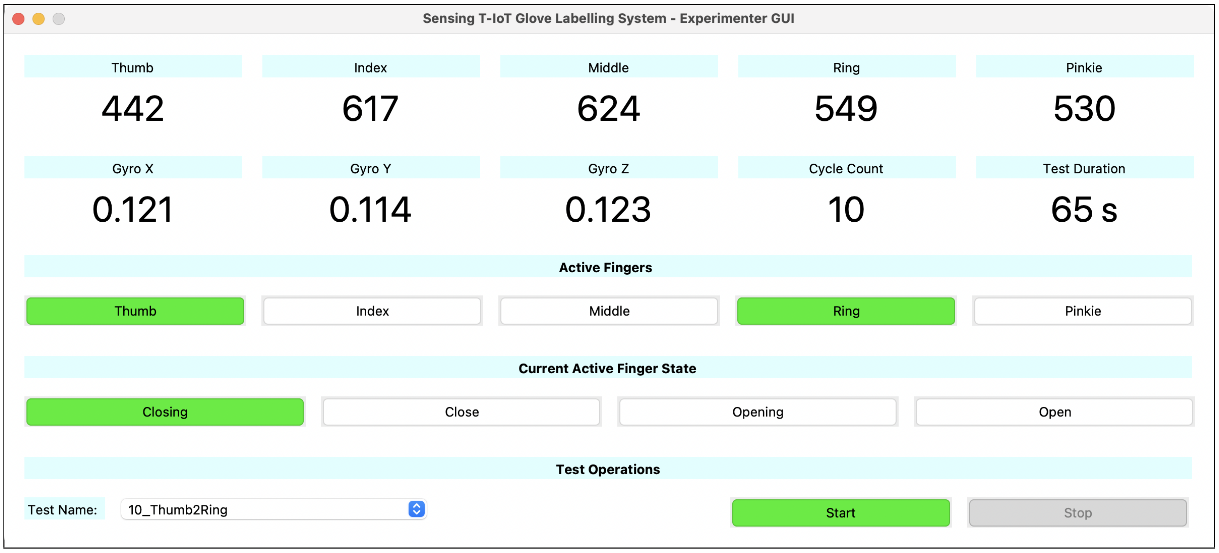

Figure S4: Experimenter Graphical User Interface of the Sensing T-IoT Glove Data Acquisition and Labelling System. Row 1: Instantaneous analog values of finger capacitive sensors. Row 2: Columns 1-3 display the X, Y, and Z gyroscope values of the glove used to calculate the orientation of the glove (these values are not utilized in this study). Column 4 shows the cycle count of the current test, which increments when the state changes from Open to Closing. Column 5 shows the duration of the test. Row 3: Indicates the active/moving status of test fingers—Thumb, Index, Middle, Ring, and Pinkie (Green: Active, White: Passive)—and is arranged according to the test name. Row 5: Test Operations; left: Test name, middle: Test Start Button, and right: Test Stop Button.

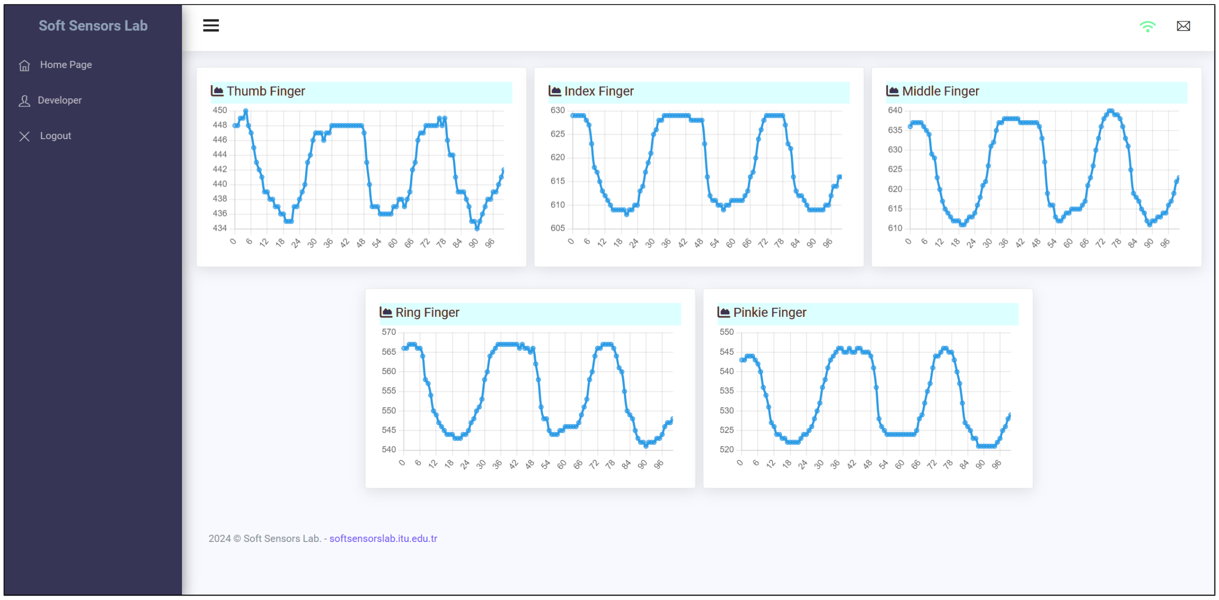

Figure S5: T-IoT Glove Finger Sensor Virtualization Interface.

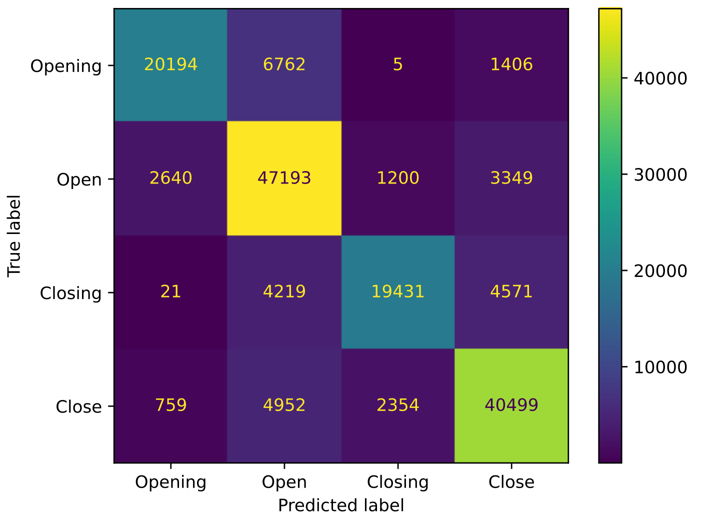

(a) Thumb Finger.

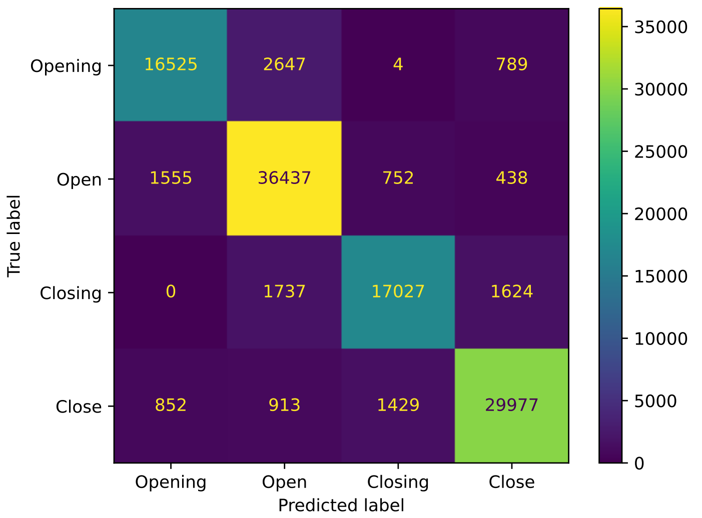

(b) Index Finger.

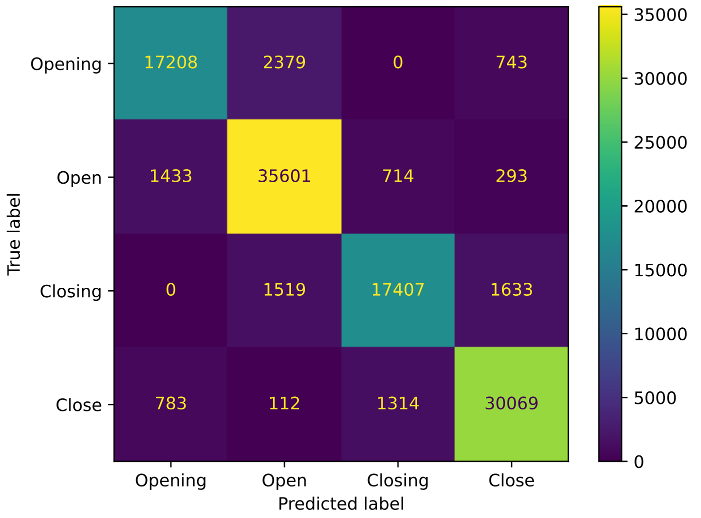

(c) Middle Finger.

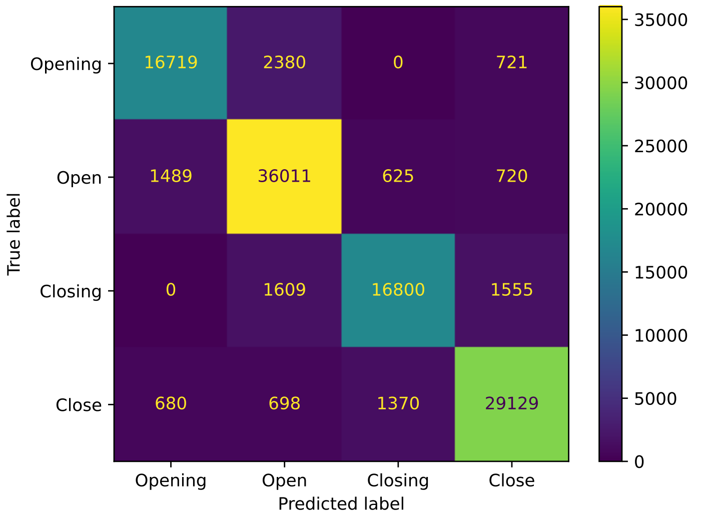

(d) Ring Finger.

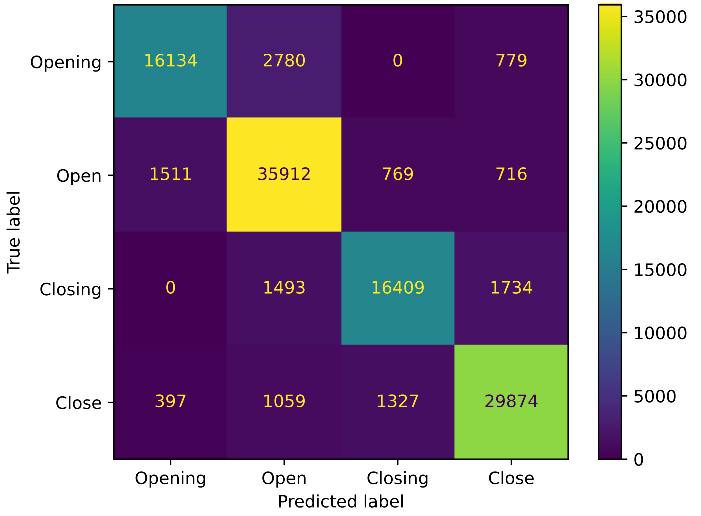

(e) Pinkie Finger.

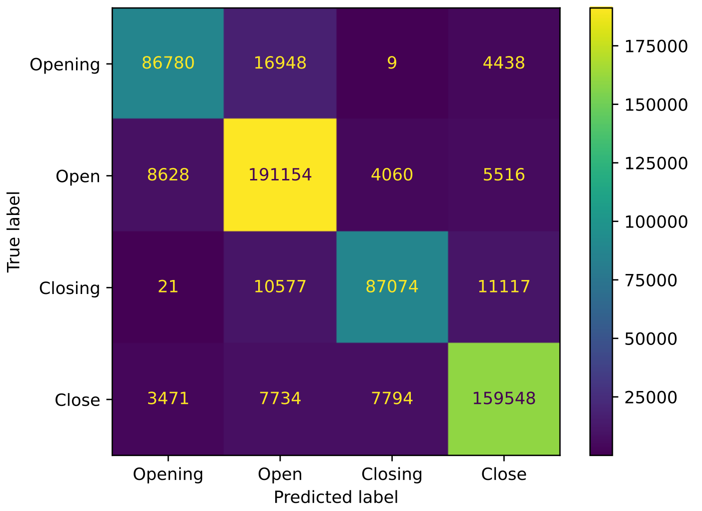

(f) Overall Results.

Figure S6: Confusion Matrices of Logistic Regression Classifier for Different Fingers.

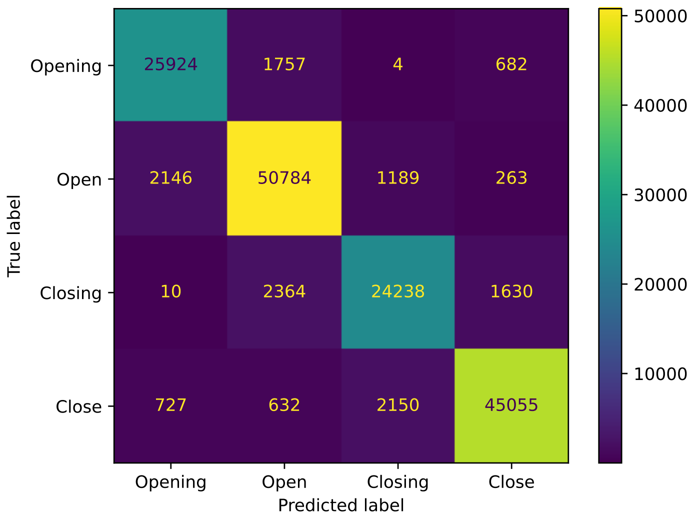

(a) Thumb Finger.

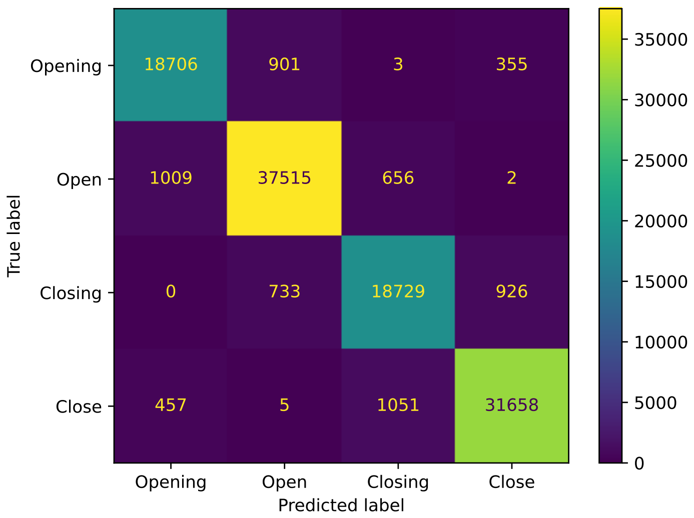

(b) Index Finger.

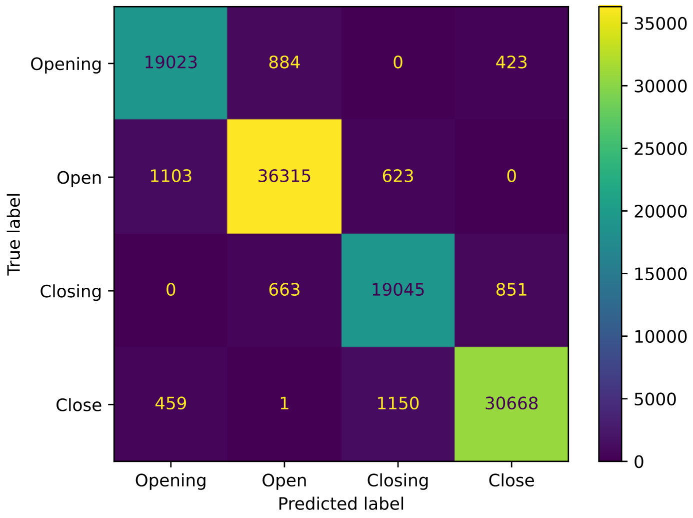

(c) Middle Finger.

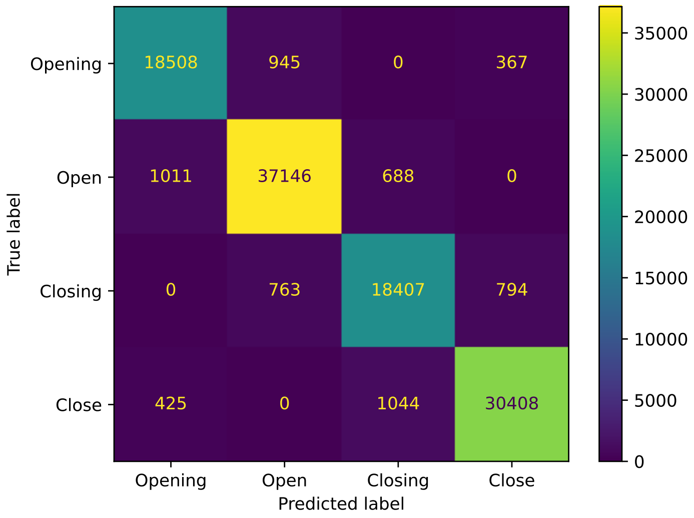

(d) Ring Finger.

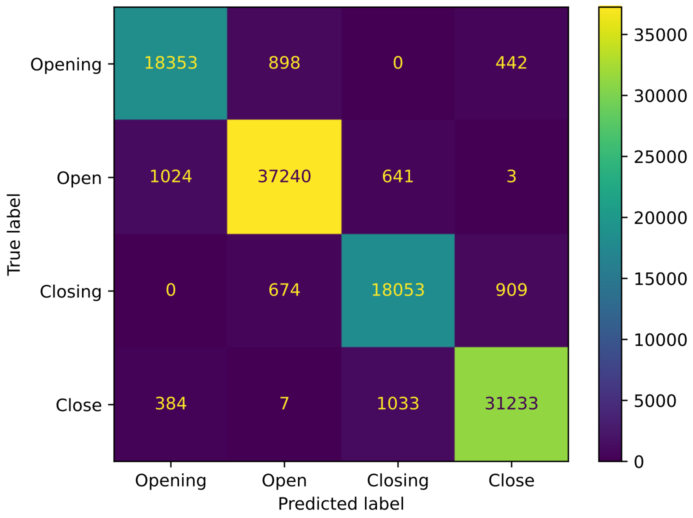

(e) Pinkie Finger.

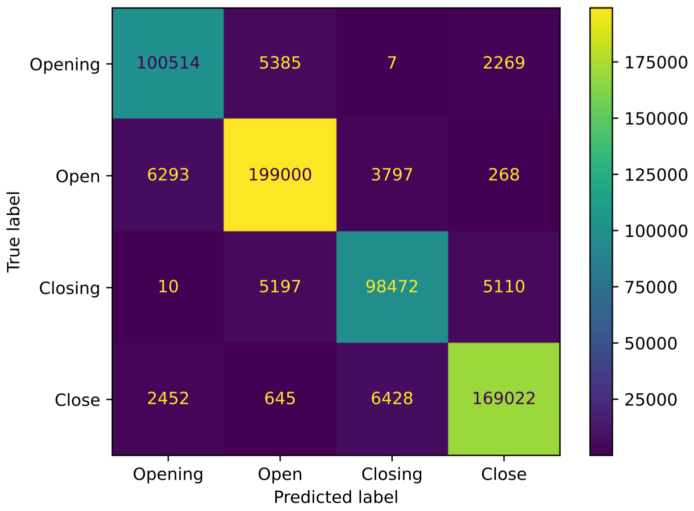

(f) Overall Results.

Figure S7: Confusion Matrices of Decision Tree Classifier for Different Fingers.

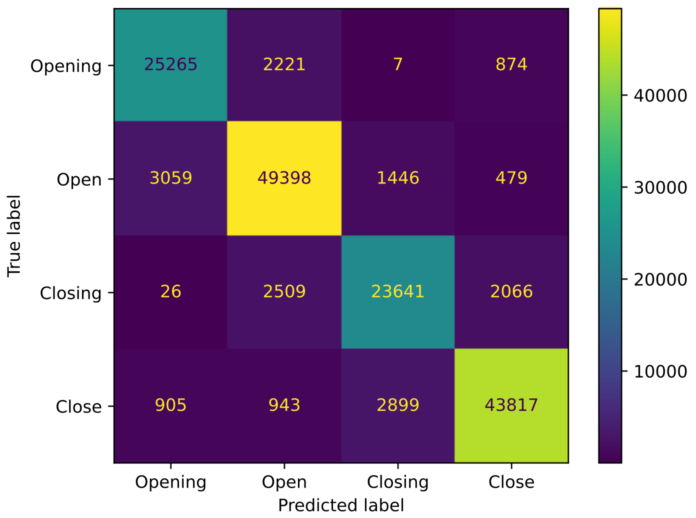

(a) Thumb Finger.

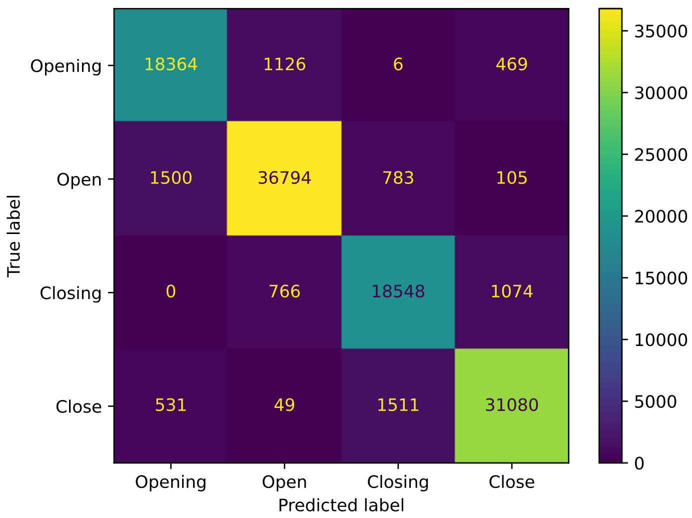

(b) Index Finger.

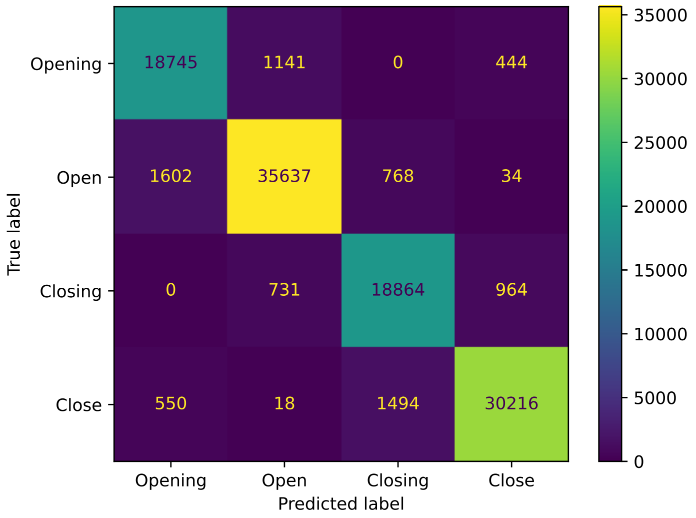

(c) Middle Finger.

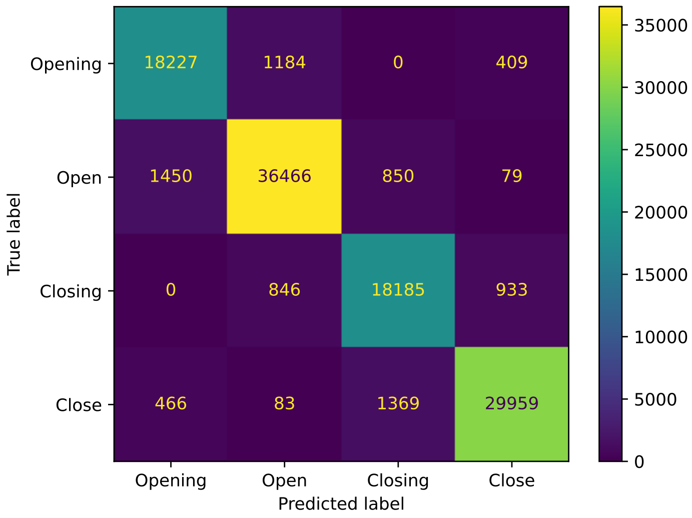

(d) Ring Finger.

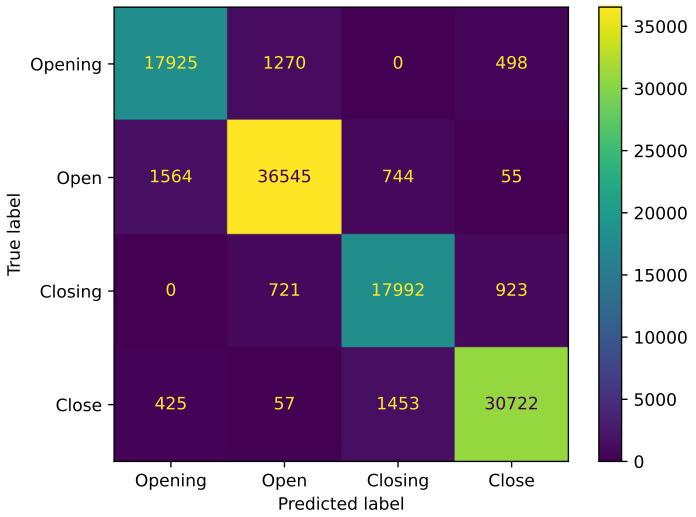

(e) Pinkie Finger.

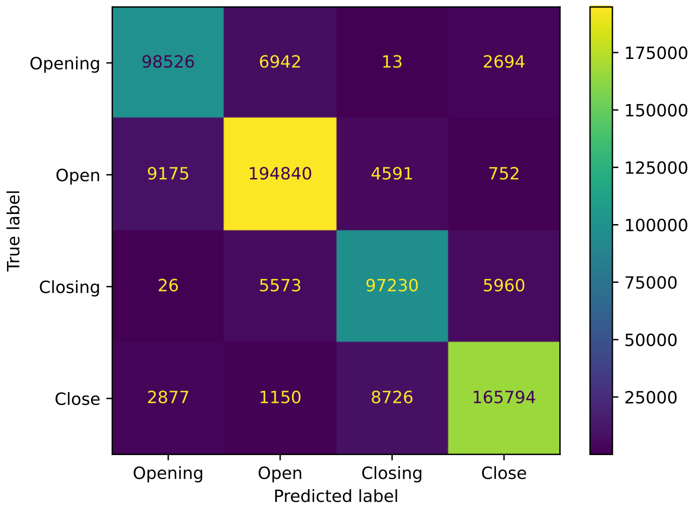

(f) Overall Results.

Figure S8: Confusion Matrices of K-Nearest Neighbors Classifier for Different Fingers.

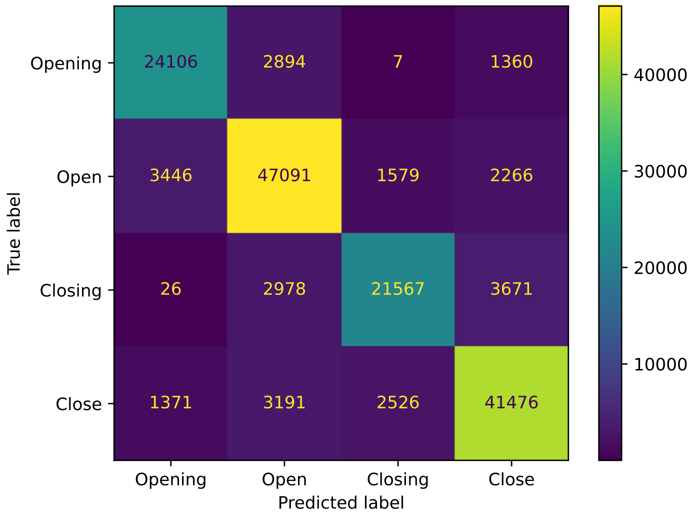

(a) Thumb Finger.

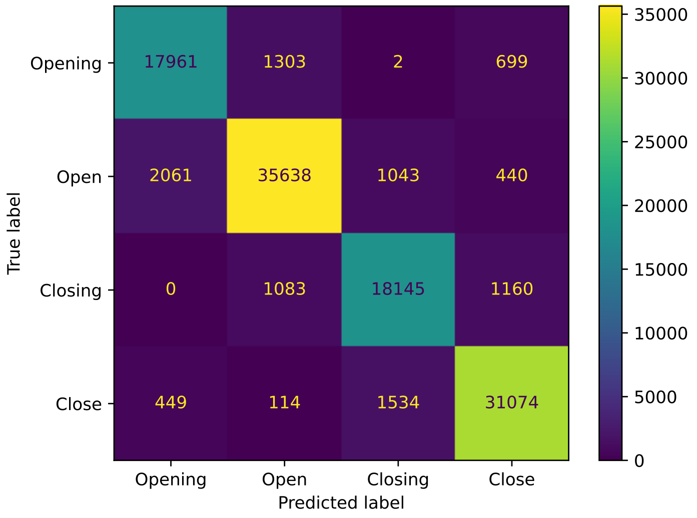

(b) Index Finger.

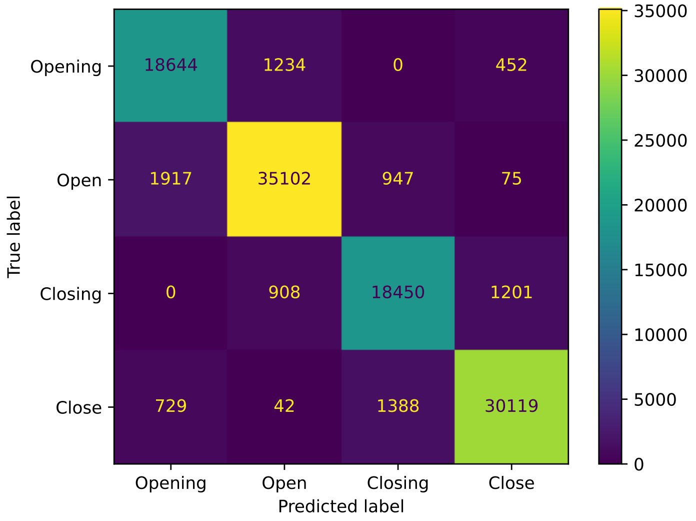

(c) Middle Finger.

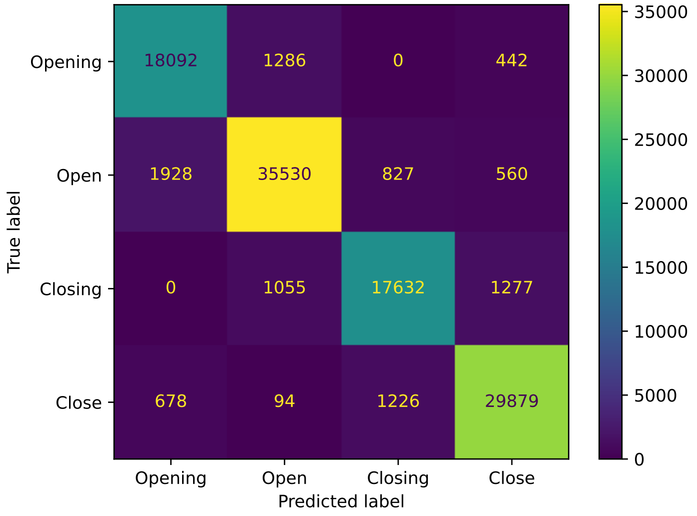

(d) Ring Finger.

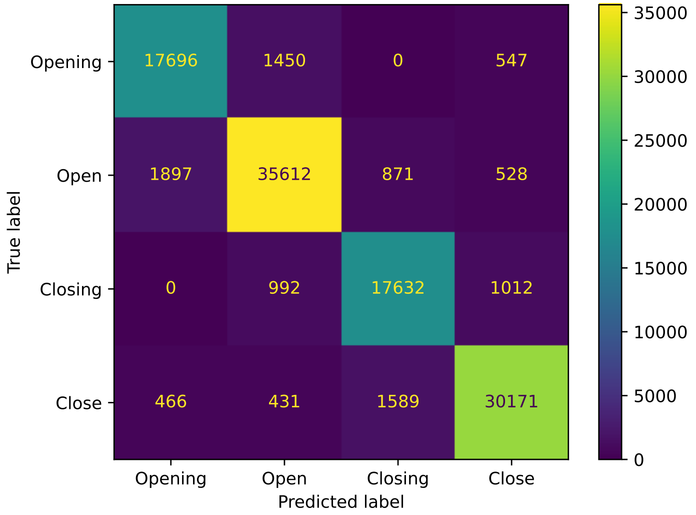

(e) Pinkie Finger.

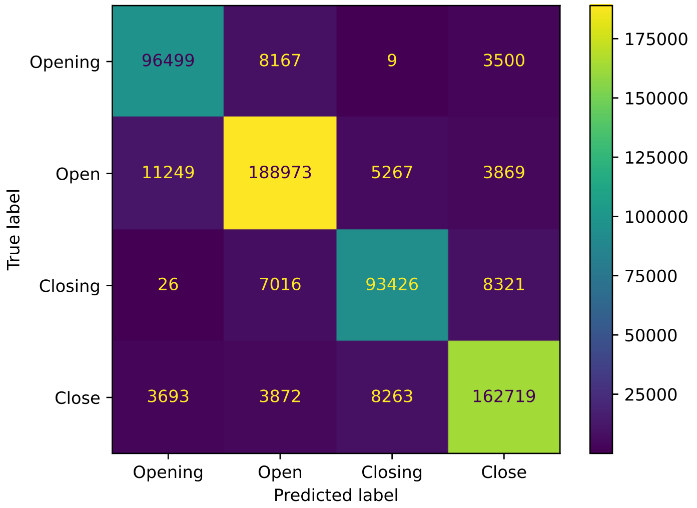

(f) Overall Results.

Figure S9: Confusion Matrices of Multi-layer Perceptron Classifier for Different Fingers.

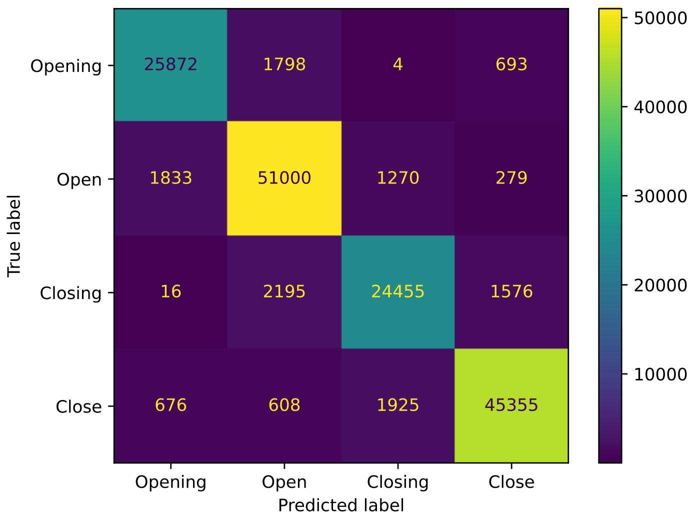

(a) Thumb Finger.

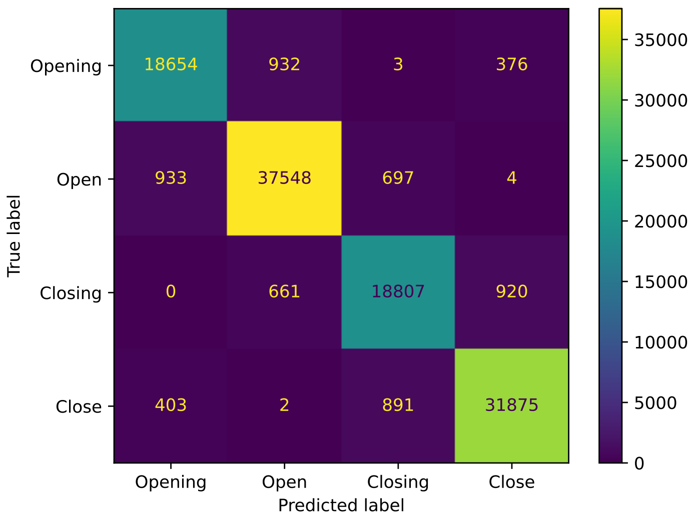

(b) Index Finger.

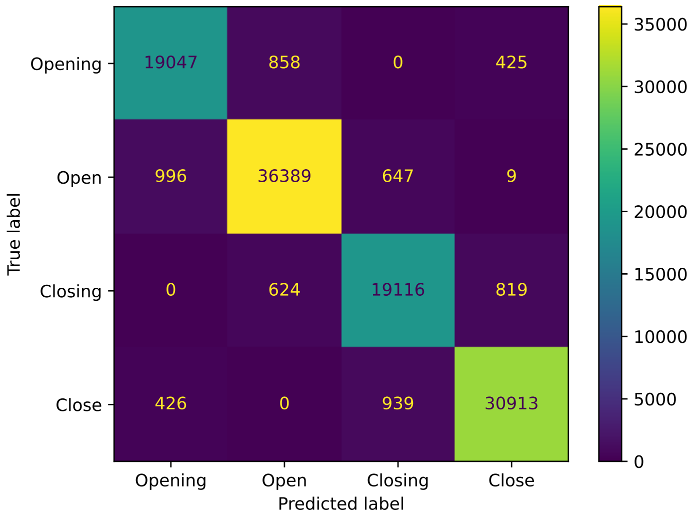

(c) Middle Finger.

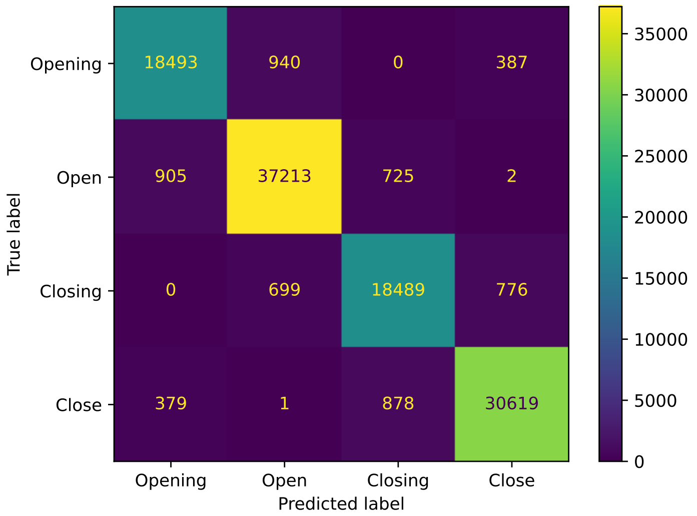

(d) Ring Finger.

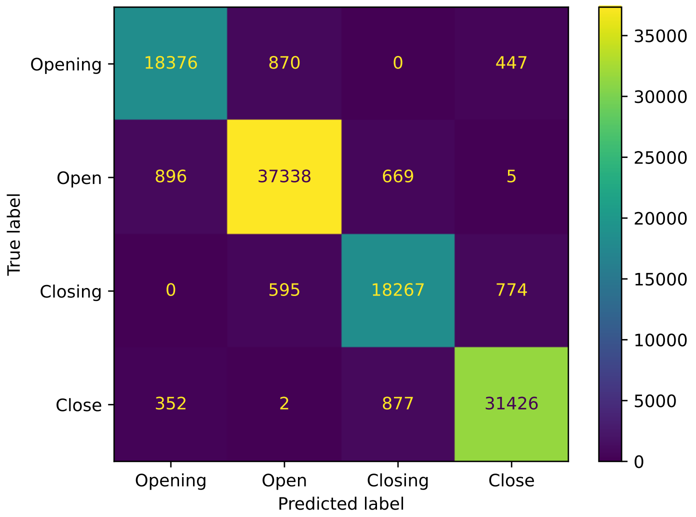

(e) Pinkie Finger.

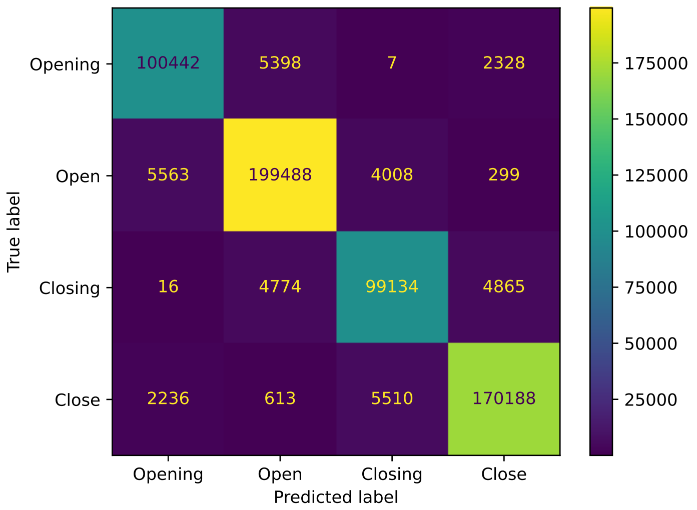

(f) Overall Results.

Figure S10: Confusion Matrices of XGBoost Classifier for Different Fingers.

Table S1. Comparison of Related Work and Proposed Study

Study	Main Application	Communication Type	Computing Platform	Sensor (master)	Control Interface	Actuator (slave)	Actuator Material Type	Actuation Type	Motion Type
[1]	Robotic hands and virtual reality (VR)	Bluetooth	Local	Wireless smart glove	Hand gesture signals - resistive sensing fibers	Rigid robotic hands	Rigid	Servo motors	Five degrees of freedom
[2]	Assists quadriplegic patients with impaired hand function	RF	Local	Wireless remote controller	Four control keys that the user can press to trigger actions	Soft robotic glove	Silicone	Pneumatic	Finger flexion and extension
[3]	Hand assistance and at-home rehabilitation	Wired	Local	Nonlinear closed-loop controller	Open and close valves manually	Soft robotic glove	Silicone	Hydraulic	Finger flexion and extension
[4]	Assitive ADLs	Wi-Fi	Local	Glove is equipped with flex sensors	Five flex sensors	Rigid robotic hand	3D printed	Tendon-driven	Mimics the finger movements
[5]	Hand therapy for rehabilitation	Wired	Local	GUI	GUI	Semi-rigid glove	Silicone and semi rigid blocks	Pneumatic	Bending motion, finger flexion and extension.
[6]	HR	Wi-Fi	Local	IMU glove	IMU sensors on each finger	Rigid robotic glove	3D printed	Servo motors	Flexion and extension
[7]	Rehabilitation and assistance in ADL	Wired	Close loop system	sEMG	sEMG	Soft robotic glove	Fabric	Pneumatic	Bending
[8]	Assisting ADLs	Wired	Local	Web-based interface	Web-based interface	Soft robotic glove	Fabric	Pneumatic	Flexion and extension
[9]	HR & AG	Bluetooth	Local	Smartphone application	Smartphone application	Soft robotic glove	Silicon and PE Tendons	Tendon-driven and pneumatic	Finger abduction and thumb extension
[10]	HR & AG	Wired	Local closed loop system	Control system	From Computer	Soft robotic glove	Silicone and fabric	Pneumatic	Bending and rotating actuators
[11]	Rehabilitation	Bluetooth	Hybrid model combining Neural Network	sEMG	sEMG	Soft robotic glove	Silicone	Pneumatic	Extension, rest, spherical grip, and fist

[12]	Augment grasp capability and provide assistive functionality	Wired	Local	Thumb-driven joystick	Thumb-driven joystick	Soft robotic glove	Fabric and bowden cables	Tendon-driven	Grasping
[13]	*Assisting AOL*	Bluetooth	Local	Smart phone	Smart phone	Soft robotic glove	TPU	*Pneumatic*	Flexion and extension
[14]	Assistive functionality and rehabilitation support	Bluetooth	Local closed loop	Smart phone	Smart phone	Soft robotic glove	TPU fabric and PE tendons	Tendon-driven and pneumatic	Flexion and extension
[15]	Rehabilitation	Wired	Local	Leap motion sensor	Hand's movements	Soft robotic glove	TPU	Pneumatic	Flexion and extension
[16]	Assistance and rehabilitation	Wired connections	Local	Hardware for control and monitoring	Touchscreen	Soft robotic glove	Silicone	Pneumatic	Flexion and extension
[17]	Rehabilitation	Wired	Local closed loop	EEG cap	EEG signals	Soft robotic glove	Fabric	Pneumatic	Flexion and extension
[18]	Telerehabilitation	Bluetooth	Cloud computing	Tablet interface	Tablet interface	Soft robotic glove	Textile and TPU	Pneumatic	Finger flexion and extension
[19]	Rehabilitation	Bluetooth	Local	Leap motion	Hand movement	Soft robotic glove	Silicone	Pneumatic	Flexion and extension
[20]	Rehabilitation	Wired	Closed loop model	Controller board	Manual air pressure control	Soft robotic glove	Fiber and Silicone	Pneumatic	Flexion and extension
[21]	Rehabilitation	Wired	Local	EEG-based Brain-Machine Interface	EEG signals	Soft robotic glove	3D printed and polyester based fabric	Tendon-driven	Flexion and extension
[22]	Assisting	Wirelessly	Local	Tablet computer and wearable EEG cap	EEG and EOG	Rigid Glove	Titanium alloy cable and sheats	DC electric motors	Opening and closing
[23]	Home-based rehabilitation	Wired	Local	Manual adjustment of solenoid valves	Predetermined pattern	Soft robotic glove	Textile	Pneumatic	Flexion and extension
[24]	Rehabilitation	Bluetooth	Local	sEMG	sEMG and smartphone application	Soft robotic glove	3D printed TPU	Tendon-driven	Flexion and extension
[25]	Assisting	Bluetooth	Direct control	External control interface	External control interface	Soft robotic glove	Fabric	Pneumatic	Finger flexion and extension
[26]	Assisting rehabilitation	Local	Local	Glove's control box	User-controlled button	Soft robotic Glove	Textile and TPE	Pneumatic	Finger flexion and extension

[27]	Rehabilitation assistance	Local	Local	Pneumatic valves and control units	Local pneumatic control	Soft robotic glove	Fabric and Elastomer	Pneumatic	Finger flexion and extension
[28]	Assistive	Wired	Local	Pneumatic pressure regulator.	Button	Fabric robotic device	Fabric	Pneumatic	Finger flexion and extension
[29]	Teleoperation	Wi-Fi	Cloud Computing	EMG and IMU	EMG and IMU	Robotic arm and hand	Rigid	Motors	Bending and grasping
[30]	Remote bomb defusal robot, healthcare, remote assistance	Bluetooth	Cloud computing	Smart glove with graphene/Ecoflex strain sensors	Hand gestures	Robots	Rigid	Motors	Forward moving, back moving, left turning, right turning, and the robotic arm's movements
[31]	Home-based rehabilitation	Wired	Local	Inner glove with resistive sensors into Actuating glove	Human-computer interface	Soft glove	Soft	Pneumatic	Flexion, extension, and bending
[32]	Rehabilitation for assisting	Wired	Local	GUI	Button-controlled	Robotic glove	TPU coated fabric	Pneumatic	Flexion and extension
[33]	HR	Local- Bluetooth	Local	Smartphone application	Speech recognition	Soft exoskeleton glove	Soft	Tendon driven	Flexion and extension
[34]	Teleoperation surgery and rescue operations	5G	Edge computing – Neural Network	Haptic glove	Angular parameters and torques observation	Robotic hand	Rigid	Motor	Grasping and lifting
[35]	Rehabilitation	Internally	Internally	Control unit	Touch sensors	Soft robotic assistive device	Soft - Textile	Artificial tendons	Grip control
[36]	Rehabilitation	Local	Local	Hand-and-wrist sensor glove	Hand motion	Soft hand and wrist exoskeleton	Silicone	Pneumatic	Flexion and extension
[37]	Assistive rehabilitation	Bluetooth	Neural network	Myo Armband sEMG	sEMG	Soft Exo-Glove	Soft	Shape Memory Alloy(SMA)-actuated tendons	Grasping
Proposed work	Telerehabilitation	Bluetooth	Cloud Computing	Sensing T-IoT Glove	Capacitive Sensors	Actuating T-IoT Glove	Soft, Textile, TPU Bladder	Pneumatic	Flexion and extension

Table S2. Abbreviations of Table 1

**ADL**	Activities of Daily Living
**AG**	Assistive Grasping
**EEG**	ElectroEncephaloGraphy
**EMG**	ElectroMyoGraphy
**EOG**	ElectroOculoGraphy
**GUI**	Graphical User Interface
**HR**	Hand Rehabilitation
**PE**	PolyEthylene
**RF**	Radio Frequency
**sEMG**	Surface ElectroMyoGraphy
**TPE**	Thermoplastic Elastomer
**TPU**	Thermoplastic PolyUrethane
**VR**	Virtual Reality

References

[1] W. Gu, S. Yan, J. Xiong, Y. Li, Q. Zhang, K. Li, C. Hou, H. Wang, *Chem. Eng. J.* **2023**, *460*, 141777.

[2] Z. Jiryaei, A. A. Alvar, M. A. Bani, M. Vahedi, A. S. Jafarpisheh, N. Razfar, *J. Bodyw. Mov. Ther.* **2021**, *27*, 731.

[3] P. Polygerinos, Z. Wang, K. C. Galloway, R. J. Wood, C. J. Walsh, *Robot. Auton. Syst.* **2015**, *73*, 135.

[4] F. Salman, Y. Cui, Z. Imran, F. Liu, L. Wang, W. Wu, *Sens. Actuators Phys.* **2020**, *309*, 112004.

[5] M. Haghshenas-Jaryani, R. M. Patterson, N. Bugnariu, M. B. J. Wijesundara, *J. Hand Ther.* **2020**, *33*, 198.

[6] T. Triwiyanto, S. Luthfiyah, I. Putu Alit Pawana, A. Ali Ahmed, A. Andrian, *HardwareX* **2023**, *14*, e00432.

[7] Y. Chen, X. Tan, D. Yan, Z. Zhang, Y. Gong, *IEEE J. Transl. Eng. Health Med.* **2020**, *8*, 1.

[8] J. C. Maldonado-Mejía, M. Múnera, C. A. R. Diaz, H. Wurdemann, M. Moazen, M. J. Pontes, M. E. Vieira Segatto, M. E. Monteiro, C. A. Cifuentes, *Front. Neurorobotics* **2023**, *17*, 1091827.

[9] L. Gerez, G. Gao, A. Dwivedi, M. Liarokapis, *IEEE Access* **2020**, *8*, 173345.

[10] Y. Zhu, W. Gong, K. Chu, X. Wang, Z. Hu, H. Su, *Sensors* **2022**, *22*, 6294.

[11] Y. Chen, Z. Yang, Y. Wen, *Sensors* **2021**, *21*, 578.

[12] R. Alicea, M. Xiloyannis, D. Chiaradia, M. Barsotti, A. Frisoli, L. Masia, *Wearable Technol.* **2021**, *2*, e4.

[13] J. Lai, A. Song, J. Wang, Y. Lu, T. Wu, H. Li, B. Xu, X. Wei, *IEEE Trans. Neural Syst. Rehabil. Eng.* **2023**, *31*, 3223.

[14] H. Liu, C. Wu, S. Lin, Y. Chen, Y. Hu, T. Xu, W. Yuan, Y. Li, *Adv. Intell. Syst.* **2023**, *5*, 2200274.

[15] J. Lai, A. Song, Y. Li, Y. Lu, K. Shi, in *Proc. 2023 9th Int. Conf. Robot. Artif. Intell.*, ACM, Singapore Singapore, **2023**, pp. 1–6.

[16] K. H. L. Heung, H. Li, Thomson. W. L. Wong, S. S. M. Ng, *Front. Bioeng. Biotechnol.* **2023**, *11*, 1188996.
[17] N. Cheng, K. S. Phua, H. S. Lai, P. K. Tam, K. Y. Tang, K. K. Cheng, R. C.-H. Yeow, K. K. Ang, C. Guan, J. H. Lim, *IEEE Trans. Biomed. Eng.* **2020**, *67*, 3339.
[18] T. Proietti, K. Nuckols, J. Grupper, D. Schwerz De Lucena, B. Inirio, K. Porazinski, D. Wagner, T. Cole, C. Glover, S. Mendelowitz, et al., *Wearable Technol.* **2024**, *5*, e1.
[19] J. Lai, A. Song, K. Shi, Q. Ji, Y. Lu, H. Li, *IEEE Trans. Med. Robot. Bionics* **2023**, *5*, 730.
[20] K. Ma, Z. Jiang, S. Gao, X. Cao, F. Xu, *IEEE Robot. Autom. Lett.* **2022**, *7*, 6115.
[21] R. S. Araujo, C. R. Silva, S. P. N. Netto, E. Morya, F. L. Brasil, *Front. Neurosci.* **2021**, *15*, 661569.
[22] S. R. Soekadar, M. Witkowski, C. Gómez, E. Opisso, J. Medina, M. Cortese, M. Cempini, M. C. Carrozza, L. G. Cohen, N. Birbaumer, et al., *Sci. Robot.* **2016**, *1*, eaag3296.
[23] K. Nuckols, C. J. Hohimer, C. Glover, D. S. De Lucena, W. Moyo, D. Wagner, A. Cloutier, D. J. Lin, C. J. Walsh, in *2020 8th IEEE RASEMBS Int. Conf. Biomed. Robot. Biomechatronics BioRob*, IEEE, New York City, NY, USA, **2020**, pp. 428–433.
[24] A. Mohammadi, J. Lavranos, P. Choong, D. Oetomo, in *2018 40th Annu. Int. Conf. IEEE Eng. Med. Biol. Soc. EMBC*, IEEE, Honolulu, HI, **2018**, pp. 2120–2123.
[25] M. Feng, D. Yang, G. Gu, *IEEE Robot. Autom. Lett.* **2021**, *6*, 3105.
[26] C. Correia, K. Nuckols, D. Wagner, Y. M. Zhou, M. Clarke, D. Orzel, R. Solinsky, S. Paganoni, C. J. Walsh, *IEEE Trans. Neural Syst. Rehabil. Eng.* **2020**, *28*, 1407.
[27] J. Wang, Z. Liu, Y. Fei, *J. Mech. Robot.* **2019**, *11*, 011015.
[28] C. Suulker, A. Greenway, S. Skach, I. Farkhatdinov, S. Charles Miller, K. Althoefer, *IEEE Robot. Autom. Lett.* **2024**, *9*, 7811.
[29] M. Chu, Z. Cui, A. Zhang, J. Yao, C. Tang, Z. Fu, A. Nathan, S. Gao, *IEEE Internet Things J.* **2022**, *9*, 19717.
[30] J. Zhou, X. Long, J. Huang, C. Jiang, F. Zhuo, C. Guo, H. Li, Y. Fu, H. Duan, *Npj Flex. Electron.* **2022**, *6*, 55.
[31] F. Meng, C. Liu, Y. Li, H. Hao, Q. Li, C. Lyu, Z. Wang, G. Ge, J. Yin, X. Ji, et al., *Electronics* **2023**, *12*, 2531.
[32] H. K. Yap, P. M. Khin, T. H. Koh, Y. Sun, X. Liang, J. H. Lim, C.-H. Yeow, *IEEE Robot. Autom. Lett.* **2017**, *2*, 1383.
[33] P. Tran, S. Jeong, S. L. Wolf, J. P. Desai, *IEEE Robot. Autom. Lett.* **2020**, *5*, 898.
[34] M. Ahmed, L. Daksha, V. Kahar, N. Mahavar, Q. Abbas, R. Kumar, A. Kherani, B. Lall, *Wirel. Pers. Commun.* **2024**, DOI 10.1007/s11277-024-10872-3.
[35] A. I. R. Kottink, C. D. M. Nikamp, F. P. Bos, C. K. V. D. Sluis, M. V. D. Broek, B. Onneweer, J. M. . Stolwijk-Swüste, S. M. Brink, N. B. M. Voet, J. S. Rietman, et al., *PLOS ONE* **2024**, *19*, e0306713.
[36] T. Ridremont, I. Singh, B. Bruzek, V. Erel, A. Jamieson, Y. Gu, R. Merzouki, M. B. J. Wijesundara, *Machines* **2024**, *12*, 288.
[37] D. Copaci, D. S. D. Cerro, J. A. Guadalupe, L. M. Lorente, D. B. Rojas, *IEEE Access* **2024**, *12*, 43506.
